# Genome and Proteome Analysis of *Rhodococcus erythropolis* MI2: Elucidation of the 4,4´-Dithiodibutyric Acid Catabolism

**DOI:** 10.1371/journal.pone.0167539

**Published:** 2016-12-15

**Authors:** Heba Khairy, Christina Meinert, Jan Hendrik Wübbeler, Anja Poehlein, Rolf Daniel, Birgit Voigt, Katharina Riedel, Alexander Steinbüchel

**Affiliations:** 1 Institut für Molekulare Mikrobiologie und Biotechnologie, Westfälische Wilhelms-Universität Münster, Münster, Germany; 2 Botany and Microbiology Department, Faculty of Science, Alexandria University, Alexandria, Egypt; 3 Department of Genomic and Applied Microbiology and Göttingen Genomics Laboratory, Institut für Mikrobiologie und Genetik, Georg-August-Universität Göttingen, Göttingen, Germany; 4 Institut für Mikrobiologie, Ernst-Moritz-Arndt Universität, Greifswald, Germany; 5 Faculty of Environmental Sciences, King Abdulaziz University, Jeddah, Saudi Arabia; National Renewable Energy Laboratory, UNITED STATES

## Abstract

*Rhodococcus erythropolis* MI2 has the extraordinary ability to utilize the xenobiotic 4,4´-dithiodibutyric acid (DTDB). Cleavage of DTDB by the disulfide-reductase Nox, which is the only verified enzyme involved in DTDB-degradation, raised 4-mercaptobutyric acid (4MB). 4MB could act as building block of a novel polythioester with unknown properties. To completely unravel the catabolism of DTDB, the genome of *R*. *erythropolis* MI2 was sequenced, and subsequently the proteome was analyzed. The draft genome sequence consists of approximately 7.2 Mbp with an overall G+C content of 62.25% and 6,859 predicted protein-encoding genes. The genome of strain MI2 is composed of three replicons: one chromosome and two megaplasmids with sizes of 6.45, 0.4 and 0.35 Mbp, respectively. When cells of strain MI2 were cultivated with DTDB as sole carbon source and compared to cells grown with succinate, several interesting proteins with significantly higher expression levels were identified using 2D-PAGE and MALDI-TOF mass spectrometry. A putative luciferase-like monooxygenase-class F_420_-dependent oxidoreductase (RERY_05640), which is encoded by one of the 126 monooxygenase-encoding genes of the MI2-genome, showed a 3-fold increased expression level. This monooxygenase could oxidize the intermediate 4MB into 4-oxo-4-sulfanylbutyric acid. Next, a desulfurization step, which forms succinic acid and volatile hydrogen sulfide, is proposed. One gene coding for a putative desulfhydrase (RERY_06500) was identified in the genome of strain MI2. However, the gene product was not recognized in the proteome analyses. But, a significant expression level with a ratio of up to 7.3 was determined for a putative sulfide:quinone oxidoreductase (RERY_02710), which could also be involved in the abstraction of the sulfur group. As response to the toxicity of the intermediates, several stress response proteins were strongly expressed, including a superoxide dismutase (RERY_05600) and an osmotically induced protein (RERY_02670). Accordingly, novel insights in the catabolic pathway of DTDB were gained.

## Introduction

*Rhodococcus erythropolis* strain MI2 is a Gram-positive, aerobic and non-motile bacterium belonging to the family *Nocardiaceae*, which contains highly diverse genera and species [1, 2]. As rhodococci, which are known to be ubiquitous in nature, usually possess large genomes, it is not surprising that members of this genus reveal a well-established cellular resistance for many toxic compounds and marvelous metabolic abilities for the degradation of various xenobiotic compounds [3–6]. The presence of multiple paralogues participating in major catabolic pathways, in addition to the existence of large linear plasmids conferring an outstanding capacity to utilize diverse substrates, marked this genus significantly in the field of bioremediation [3, 6, 7]. Many strains of *R*. *erythropolis* are well-studied due to their broad substrate specificities, biotechnological properties, and potent adaptabilities to extreme conditions [8]. *R*. *erythropolis* strain MI2 was isolated, and later applied in polythioester (PTE) research, because this strain exhibits the rare ability to use the synthetic disulfide 4,4´-dithiodibutyric acid (DTDB) as sole carbon source and electron donor for aerobic growth [9, 10].

DTDB is an organic sulfur compound used in diverse technical applications, such as in the production of chemically crosslinked epoxidized natural rubber [11] and as an alternative monolayer for protein chips [12, 13]. Furthermore, DTDB is considered as an alternative promising substrate for PTE synthesis. PTEs show interesting material properties in comparison to their polyoxoester equivalent, particularly their non-biodegradability could make them valuable [14–18]. For the generation of *metabolically engineered* PTE-production strains, it is necessary to investigate the microbial catabolism of DTDB. This nontoxic PTE-precursor substrate could result in the formation of the hitherto unknown poly(4-mercaptobutyric acid) (poly[4MB]), which would be the first PTE consisting of 4-mercaptoalkanoate building blocks and is therefore expected to exhibit interesting new features.

**Fig 1:**
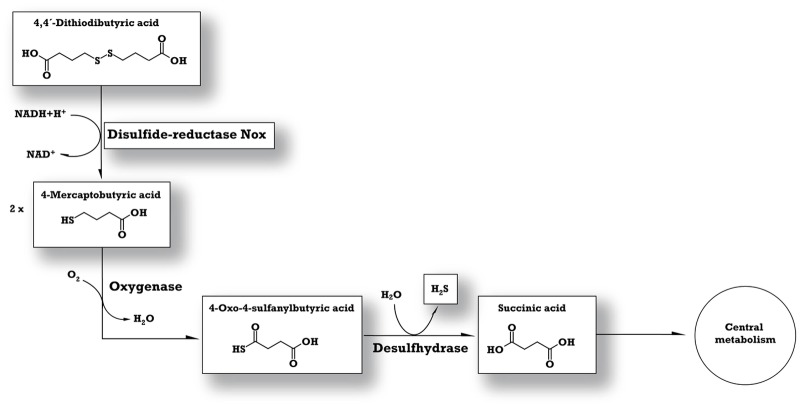
Hypothesized catabolic pathway of DTDB by *R*. *erythropolis* MI2. DTDB is cleaved into two molecules 4MB by Nox using NADH. Then, a putative oxygenase converts 4MB into 4-oxo-4-sulfanylbutyric acid, which could most probably be desulfhydrated yielding succinic acid, thereby releasing the sulfur as H_2_S. Intermediates and the enzyme emphasized in boxes have been identified in previous studies [9, 10].

In a previous study, degradation intermediates of DTDB were detected and a hypothetical pathway was postulated [9]. Afterwards, the gene/enzyme responsible for the initial cleavage of the microbial DTDB degradation was identified *via* transposon-induced mutagenesis [10]. The transposon element IS*1415* was mapped three base pairs upstream of a gene coding for the transcriptional regulator NoxR, which apparently regulates the transcription of a downstream-localized gene encoding the NADH:flavin oxidoreductase Nox [10]. Biochemical activity assays proved that Nox_MI2_ catalyses the reduction of DTDB, forming two molecules of 4MB [19]. Further steps of the 4MB-utilization were only theoretical and the suggested pathway was based solely on the detected intermediates (Fig 1). Presumably, an oxygenase converts 4MB into 4-oxo-4-sulfanylbutyric acid. Then, a putative desulfhydrase could form succinic acid and volatile hydrogen sulfide.

To further elucidate the catabolic pathway of DTDB in *R*. *erythropolis* strain MI2, the genome was sequenced. Then, it was possible to apply a proteomic approach to shed light on the role of so far unknown or non-characterized proteins, especially those involved in the fate of 4MB, but also to define interesting features of strain MI2.

## Materials and Methods

### Cultivation conditions

*Rhodococcus erythropolis* MI2 was cultivated aerobically at 30°C in lysogeny broth (LB) medium [20, 21] or in mineral salts medium (MSM) [22, 23] containing the required carbon source. All carbon sources were prepared as filter-sterilized 20% (w/v) stock solutions, and the pH was adjusted to 7.0. DTDB was added as a carbon source at a concentration of 30 mM. Solid medium contained 1.8% (w/v) purified agar-agar.

### Isolation of the genomic DNA

Genomic DNA was isolated by the DNA preparation kit Qiagen DNeasy Blood and tissues (Qiagen, Hilden, Germany).

### Plasmids isolation by pulsed-field gel electrophoresis (PFGE)

PFGE was done according to [24]. Cell pellets from 16 h incubated cultures were frozen at −80°C for > 1 h, then resuspended in 1 ml of resuspension buffer (10 mM Tris, pH 8.0, 1 mM EDTA, and 10% Triton X-100). The resuspended cells were incubated at 30°C for 2 h with gentle shaking, pelleted, washed with 1 ml wash buffer (0.2 M NaCl, 10 mM Tris, pH 8.0, and 100 mM EDTA), and pelleted again. Cell pellets were resuspended in the appropriate amount of wash buffer. This suspension was pre-heated at 42°C before it was mixed with an equal volume of molten 1% low melt agarose (Roth, Karlsruhe, Germany) in 1 X TBE, yielding a final cell concentration of 100 μg/μl. 80 μl of this mixture were quickly dispensed into a disposable plug mold (Bio-Rad). The 0.5% agarose plugs containing embedded bacterial cells were incubated at 4°C for 15 min to ensure solidification. Then, individual plugs were incubated with 1 ml of enzyme lysis solution (10 mM Tris, pH 8.0, 50 mM NaCl, and 100 mM EDTA) containing fresh lysozyme (5 mg/ml) and mutanolysin (200 U/ml) (Sigma–Aldrich Chemie Steinheim, Germany) for 2 h at 37°C in microfuge tubes with gentle shaking. The supernatant was removed, and the plugs were treated with 1 ml of detergent lysis solution (10 mM Tris, pH 8.0, 50 mM NaCl, 100 mM EDTA, 4.8 mM sodium deoxycholate, and 1.7 mM N-lauryl sarcosine) for 16 h at 37°C on a shaking platform. The supernatant was discarded and the plugs were washed once with washing buffer. Finally, the plugs were incubated with 1 ml of digestion buffer (10 mM Tris, pH 8.0, 50 mM EDTA, and 3.4 mM N-lauryl sarcosine) containing fresh proteinase K (0.5 mg/ml) for 16 h at 50°C with continuous shaking. Genomic DNA were resolved using the Bio-Rad CHEF-DR III PFGE (Bio-Rad, Richmond, USA) apparatus with 0.7% agarose pulsed field (Roth, Karlsruhe, Germany) in 1 X TBE at 14°C, 5 V/cm, included angle of 120°C for 24 h with 30 s initial and 60 s final switch times. The λ ladder PFG marker 50 kb-1000 kb (ProMega, Madison, USA) was used to estimate replicon size.

### Genome sequencing

Illumina shotgun sequencing libraries were generated from the extracted DNA, and sequencing was performed with a Genome Analyzer IIx, as recommended by the manufacturer (Illumina, San Diego, CA, USA). Sequencing resulted in 22,616,726 reads that were trimmed using Trimmomatic version 0.32 [25]. Phred 33 with a minimum read length of 50 was applied. The average length of the reads was 84.82. Five million of these reads were used for *de novo* assembly performed with the SPAdes genome assembler software version 3.5.0 [26], which resulted in 124 contigs (˃500 bp) with an overall coverage of 206-fold (determined by QualiMap version 2.1) [27].

### Gene prediction, annotation, analysis and comparative genomics

Automatic annotation was performed using the prokka annotation pipeline [28]. IMG/ER was used for manual revision of the automatic annotation [29]. All CDS putatively involved in DTDB catabolism were manually reviewed with criteria such as the presence of a ribosome-binding site and pfam domains, GC frame plot analysis, and comparison with sequences in the publicly available databases. *In silico* analyses were carried out applying annotation software tools of the IMG/ER system [30] and Artemis [31]. Prediction of signal peptides was carried out with TatP 1.0 [32] and SignalP [33] software. TMHMM [34, 35] was used for the prediction of transmembrane helices. KEGG Automatic Annotation Server (KAAS) was used to generate KEGG Orthology assignments (KO) and KEGG pathways [36]. Thereby, the bi-directional best hit method was exerted. Comparative genomics was done applying implemented software in IMG/ER system using default parameters, BLAST package and BLAST Ring Image Generator (BRIG) [37].

### Nucleotide sequence accession numbers

This Whole Genome Shotgun project has been deposited at DDBJ/ENA/GenBank under the accession LYPG00000000. The version described in this paper is version LYPG01000000.

### Growth conditions and cell harvest for proteome analysis

Cells of *R*. *erythropolis* strain MI2 were cultivated in mineral salt medium (MSM) [22, 23] containing 60 mM sodium succinate or 30 mM DTDB as carbon source in baffled flasks at 30°C and 120 rpm on a rotary shaker (New Brunswick Scientific Co., Inc., NJ, USA). Growth was monitored using a Klett-Summerson photometer (Manostat Corporation, NY, USA). 150 ml pre-cultures were cultivated for 20 h, and after normalizing the cell density mass to 0.1 at OD_600_, the 500 ml volume main cultures were inoculated. Samples for proteomic analysis were withdrawn during the exponential growth phase (harvest of 300 ml culture) and 7 h after entering the stationary phase (harvest of 200 ml culture). For cell harvest, the samples were transferred to 50 ml falcon tubes and were centrifuged for 30 min at 4°C and 7000 x *g* (Hettich Universal 320 R, Andreas Hettich GmbH & Co KG, Tuttlingen, Germany). The supernatant was discarded, and the remaining cell pellets were washed with fresh MSM without carbon source. Afterwards they were stored at −20°C until further use.

### Determination of DTDB consumption and hydrogen sulfide production

The consumption of DTDB was determined upon acid methylation of the lyophilized supernatants as described by [10]. Subsequently, samples were subjected to standard gas chromatographic analyses [38, 39]. Furthermore, detection of volatile hydrogen sulfide produced during the catabolism of DTDB was achieved using lead acetate test strips (Fluka, Steinheim, Germany) [10].

### Protein preparation for proteome analysis

Cell pellets were suspended in 20 ml disruption buffer (50 mM Tris/HCl, pH 7.4; 20 μg/ml DNase; 1 mM EDTA; 5 mg/ml lysozyme; 1 ml 50% (v/v) glycerol). Afterwards, the cells were disrupted with 1 g of 0.25–0.5 mm soda-lime glass beads (Carl Roth GmbH + Co.KG, Karlsruhe, Germany) using the Retsch mixer mill MM301 bead beater (Retsch GmbH, Haan, Germany) for five cycles of 30 s frequency for 10 min. Between each cycle, the 2 ml tubes were cooled on ice for 5 min. In order to remove unbroken cells and cell debris, disrupted cells were centrifuged at 4°C for 15 min at 10,000 x *g* (Centrifuge 5424R, Eppendorf, Hamburg, Germany). Samples of each phase were applied on an SDS-PAGE for ensuring protein extraction after measuring the protein concentration in the soluble fraction as described by [40].

For extraction and precipitation, the supernatant was treated with the same volume of 20% trichloroacetic acid (TCA) solution (20% (w/v) TCA and 20 mM dithiothreitol [DTT] dissolved in acetone), then incubated for 60 min at −20°C. The precipitated proteins were pelleted by 10 min centrifugation. Protein pellets were washed three times with 20 ml of 20 mM DTT in acetone, and finally the protein pellets were air-dried.

Prior to isoelectric focusing, the protein pellets were rehydrated overnight at 4°C in an adequate amount of rehydration buffer A (7 M urea, 2 M thiourea, 4% (w/v) 3-[(3- cholamidopropyl)dimethylammonio]-1-propanesulfonic [CHAPS], 100 mM DTT). Then, the samples were transferred to 1.5 ml reaction tubes and centrifuged at 17,000 x *g* for 5 min at room temperature in order to remove remaining unsolved proteins (Heraeus Pico 17 centrifuge, Thermo Scientific, Schwerte, Germany). The supernatant was transferred to a new 1.5 ml reaction tube, and the insoluble protein pellets were stored at −20°C.

Samples were diluted at least 1 to 20 with 1.5 M Tris/HCl buffer (pH 8.0) prior to the quantification of protein content to prevent disturbance by the high concentration of urea present in the rehydration buffer A. Afterwards, protein concentrations were measured according to [40]. Then, the volume corresponding to 1.5 mg protein was filled up with rehydration buffer A to a volume of 200 μl, and 150 μl rehydration buffer B (10 ml rehydration buffer A, 500 μl BioLyte^®^ 3/10 Ampholyte, 500 μl Triton X-100, a trace amount of bromophenol blue) were then added.

### 2D PAGE for proteome analysis

#### a. Isoelectric focusing (first dimension)

Ready Strip^™^ IPG strips (Bio-Rad, Hercules, USA) with a length of 17 cm and in the pH range from 4 to 7 were used for isoelectric focusing (IEF). First, rehydration of the strips was performed for 16 h at 20°C with the already prepared rehydrated 1.5 mg protein solutions. Each strip was then overlaid with 2 ml mineral oil and incubated under steady shaking. Afterward, the strips were moved to a focusing tray, covered with 2 ml mineral oil, and the IEF was done applying the following voltages: 250 V (250 Vh), 500 V (500 Vh), 1,000 V (1,000 Vh), 6,000 V (108,000 Vh), and 500 V (for up to 99 h).

#### b. SDS-PAGE (second dimension)

The IPG strips were equilibrated using the SDS-equilibration buffer (1.5 M Tris/HCl, pH 8.0, 6 M urea, 173 mM SDS and 5 M glycerin) and then fixed on 12.5% (w/v) SDS gels (200 mm × 200 mm × 1 mm) using the sealing solution (25 ml electrode buffer, 1% (w/v) agarose and trace amount of bromophenol blue) for SDS-PAGE as described by [41]. Next, the gels were placed in a DODECA Cell (PROTEAN^®^ plus DODECA^™^ Cell, Bio-Rad, Hercules, USA) already filled with 20 liter electrode buffer (25 mM Tris/HCl, 156 mM glycine, 0.1% (w/v) SDS). The temperature of the system was kept at 15°C, and the gels were run at 5 V per gel for 1.5 h and then at 20 V per gel for 7 h.

#### c. Gel staining and destaining

The gels were stained with staining solution (4 g Serva Blue G 250, 450 ml methanol, 90 ml 96% (v/v) acetic acid, 460 ml H_2_O_demin._) for approximately 16 h. Finally, gels were destained for 10 h in the destaining solution (825 ml methanol, 250 ml 96% (v/v) acetic acid, 1,425 ml H_2_O_demin._) before they were finally stored in 10% (v/v) acetic acid.

### The 2D gel images software-based evaluation

The evaluation and analysis of the 2D gel images were achieved using the software Delta2D 4.2 (Decodon GmbH, Greifswald, Germany). According to [42], gel images were imported and processed as recommended by the manufacturer. Three replicates each of both applied carbon sources (DTDB and succinate) were generated from samples of the exponential as well as the stationary growth phase. At least two gels from these three replicates, respectively, were subjected to the analysis. Warping of the gels was performed manually. Consequently, gels of the same treatment were fused to average images, and the automatically detected spots were manually corrected. A proteome map was generated using the fusion image comparing all gel images. The software was used for spot quantifications by calculating the corresponding volume percentage of each spot and relating it to the total protein content of the corresponding average gel. Spots with significantly different spot volumes, regarding the different carbon sources used for cultivation, could be identified by applying the analysis of variance tool (ANOVA) of Delta2D and setting a negative filter (0.5–2.0). Finally, spots were subjected to matrix-assisted laser desorption/ionization-time-of-flight-tandem mass spectrometry (MALDI-TOF-MS/MS) analysis.

### Mass spectrometry and data analysis

Protein spots were excised manually from the 2D gels and transferred to 1.5 ml reaction tubes containing 20 μl of 10% (v/v) acetic acid. The MALDI-TOF-MS/MS analysis was performed at the Institute of Microbiology of the Ernst-August-Arndt-University Greifswald (Germany) as described previously [43] using a 4800 Proteomics Analyzer (AB Sciex, Framingham, MA, USA). Mass spectrometry data were searched using the Mascot engine (version 2.1.0.4) against an *R*. *erythropolis* MI2 database containing common contaminants.

## Results and Discussion

### Genome

**Fig 2:**
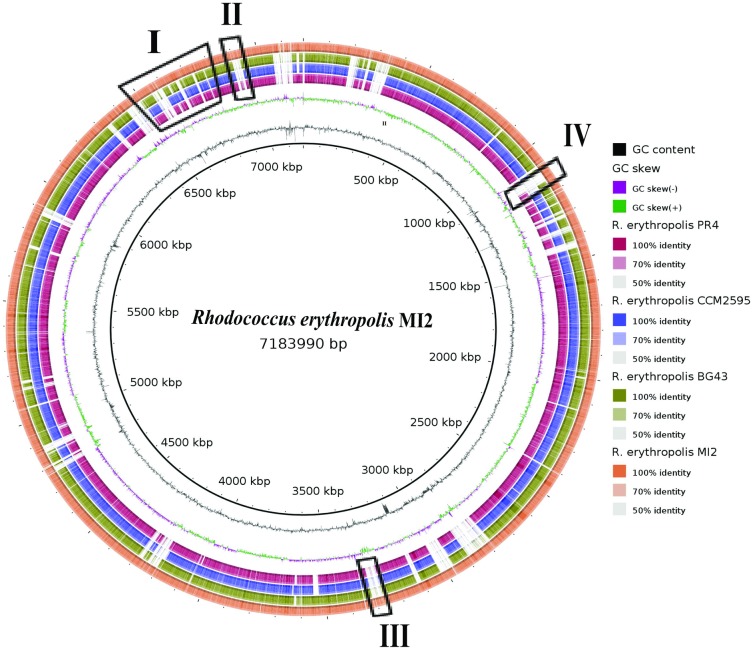
Alignment of *R*. *erythropolis* strains proteomes applying BLAST (TBLASTN) [44] and BLAST Ring Image Generator (BRIG) [37]. Strain MI2 (circle 1, orange) compared to that of strain BG43 (circle 2, green), strain CCM2595 (circle 3, blue) and strain PR4 (circle 4, red), Similarity of the proteins is symbolized by colored blocks. The more intense the color—the higher the similarity. Circles 5 and 6 represent the GC skew and GC content, respectively. Strain MI2-specific regions are highlighted by black frames labeled with roman numerals (I–IV).

The draft genome sequence of *R*. *erythropolis* strain MI2 comprises 7,183,990 bp and is composed of 6,911 predicted open reading frames (ORFs). The genome harbours 50 tRNA genes and two rRNA genes. A putative function was assigned to 5,236 (75.76%) of all predicted protein-coding sequences. The mean GC content of 62.25% is very similar to that documented for genomes of other *R*. *erythropolis* strains (Table 1). Comparative genomics using the complete genomes of *R*. *erythropolis* strain MI2, PR4, BG43 and CCM2595 revealed several unique clusters in the genome of strain MI2 (Fig 2). Clusters marked at the position “I” and “II” represent genes that could play a vital role in the degradation pathway of DTDB and will be discussed later. Additionally, the cluster at the position “III” comprises two predicted gene products (RERY_66330, RERY_66340), which were highly expressed in DTDB growing cells during the proteome analysis. Position “IV” contains a unique dioxygenase (RERY_58710) with no orthologue in other *R*. *erythropolis* strains.

**Table 1:** Genomes, number of genes, GC content, number of plasmids and Bioproject number of available completely sequenced *R*. *erythropolis* strains.

*R*. *erythropolis* strain	Genome Mbp	Number of genes	G+C content (mol%)	Number of plasmids	Bioproject number
**MI2**	7.18	6,911	62.25	2	PRJNA285438
**PR4**	6.90	6,505	62.31	3	PRJDA20395
**CCM2595**	6.37	5,899	62.45	1	PRJNA81583
**BG43**	6.87	6,506	62.28	3	PRJNA280916
**CAS922i**	7.20	6,932	62.33	*	PRJNA284797
**DN1**	6.55	6,331	62.42	*	PRJNA214035
**R138**	6.81	6,361	62.21	2	PRJNA188397
**JCM6824**	7.02	6,694	62.30	*	PRJDB3086
**SK121**	6.79	6,767	62.45	*	PRJNA34067
**XP**	7.23	6,953	62.30	*	PRJNA72225
**S-43**	6.81	8,591	62.16	*	PRJNA268657

* not determined, yet.

### Extrachromosomal elements

Most of the well-studied strains of the genus *Rhodococcus* possess one to five plasmids per strain (compare Table 1). These plasmids are either circular or linear ranging in size from 3 kbp, i.e. pREL1 from *R*. *erythropolis* PR4 to more than 1 Mbp i.e. pRHL1 from *R*. *jostii* RHA1 and account for up to 20% of the entire genome [45–48]. In the genome of *R*. *erythropolis* MI2, two megaplasmids were identified *via* PFG electrophoresis (S1 Fig). These plasmids were designated as pRLMI2-400 and pRLMI2-345 based on their approximate sizes in kbp and comprised approximately10.4% of the total genome. Searching the MI2-genome, two copies of adjacent *parAB* (RERY_03840–03830, RERY_66270–66280) in addition to a single *parB* gene (RERY_09840) were detected. Furthermore, a putative replication protein (RERY_69100) was identified. After *in silico* analyses with all available *R*. *erythropolis* genome sequences, RERY_69100 showed highest sequence similarities of 39% identical amino acids to exclusively one replicase-encoding gene (BAE46344) from *R*. *erythropolis* PR4. However, none of the 124 contigs can readily be linked to the two megaplasmids.

### Cultivation conditions for proteome analyses

To obtain sufficient cell material for the proteome analysis, cells of *R*. *erythropolis* MI2 were cultivated in MSM containing 60 mM succinate or 30 mM DTDB (S2 Fig). For each cultivation, two samples were withdrawn: (i) in the exponential growth phase at approximately 450 Klett units (KU), and (ii) 7 h after the cells had entered the stationary growth phase at approximately 650–700 KU (S2 Fig). Additionally, the consumption of DTDB was determined (S2 Fig). During the lag phase and the very early exponential phase (0–12 h), the DTDB concentration was constant. The consumption increased successively after entering the exponential phase, as cells consumed about 10 mM DTDB within 12 h of the exponential growth phase. Finally, 7 h after entering the stationary phase (directly before harvesting) the final DTDB concentration in the growth medium was 15 mM (S2 Fig). After cell disruption, samples of each phase were loaded on an SDS-PAGE to ensure the extraction of proteins (S3 Fig).

### Identification of interesting proteins during cultivation with DTDB

The focus of the proteome analyses was on proteins that showed at least a 3-fold increase in the spot volume of cells cultivated with DTDB in comparison to cells cultivated with succinate (DTDB/Succ). The dual-channel images marked with all identified spots (DTDB/Succ) are illustrated as supplemental material (S4 and S5 Figs). Moreover, the complete lists of the identified proteins including their specifications, the corresponding locus-tags, and protein ratios based on the spot volumes of the proteins spots in gels of both conditions are listed in the supplemental material (S1 and S2 Tables for proteins of the exponential phase and S3 and S4 Tables for proteins of the stationary phase, S5 Table illustrating all spot volumes of all gels).

**Fig 3:**
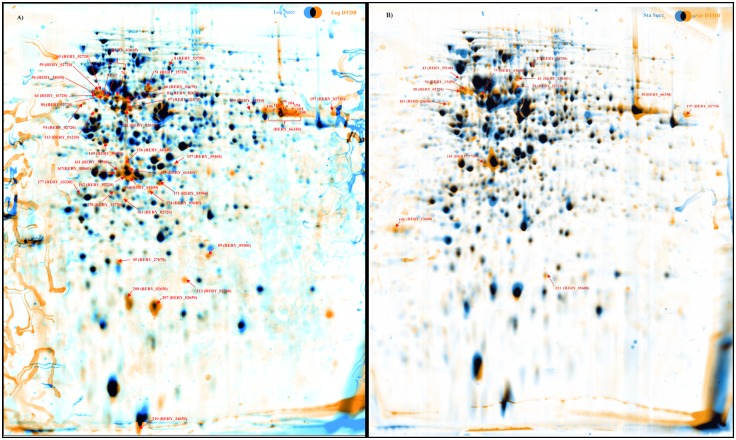
Duel channel images of both exponential and stationary phase. Dual channel images were generated applying the software Delta 2D 4.2 showing differences in the proteome of *R*. *erythropolis* MI2 cells cultivated with DTDB or succinate as revealed by 2D PAGE. In total, 1.5 mg protein was loaded onto 17 cm IPG strips with a pH range of 4–7. **A)** Cells of *R*. *erythropolis* MI2 grown with succinate (blue spots) or DTDB (orange spots), both from the exponential growth phase. **B)** Cells of *R*. *erythropolis* MI2 grown with succinate (blue spots) or DTDB (orange spots), both from the stationary phase. Black spots in A and B represent equally expressed proteins. Spots with significantly increased expression during the growth with DTDB by a factor ≥ 3 and successfully identified by MALDI-TOF-MS/MS are represented by arrows with locus tags.

Applying the Delta 2D 4.2 software (Decodon GmbH, Greifswald, Germany) 1055 protein spots were detected in total in the fused image consisting of all gel images prepared from samples of the exponential and stationary growth phase. After ANOVA analysis, 213 spots complied with the requirements of the statistical procedure and were taken into consideration for further analysis. In cells grown with DTDB, 60 spots showed significantly increased spot volumes (protein ratio > 2) in comparison to cells grown with succinic acid. Finally, 164 spots were successfully identified *via* MALDI-TOF-MS/MS analyses and could be affiliated to specific proteins encoded by the MI2 genome. The dual-channel images (Fig 3) illustrate an overlay of the average gels of DTDB-grown cells and succinate-grown cells, indicating interesting spots in the exponential (Fig 3A) and stationary phase (Fig 3B). In addition, Tables 2 and 3 list all proteins with increased spot volumes (DTDB/Succ) by a factor of more than 3 in both growth phases. The majority of proteins with high expression levels are involved in energy production and conversion, amino acid transport and lipid metabolism (Tables 2 and 3). Interestingly, some of these proteins are MI2-specific, as comparative genome analysis showed no orthologues in genomes of other *R*. *erythropolis* strains.

**Table 2:** Proteins exhibiting significantly increased expression of ≥ 3 during exponential growth phase in cells of *R*. *erythropolis* strain MI2 cultivated with DTDB (D) in comparison to cells cultivated with succinate (S).

Spot	Protein identity	Gene	ORF (RERY xxxxx)	Ratio D/S
**113**	Acetamidase/formamidase	*amdA / fmdA*	01230	5.5
**207**	Putative OsmC-like protein	-	02650	7.1
**208**	Putative OsmC-like protein	-	02650	3.5
**81**	Putative flavin amine oxidase	-	02670	5.6
**102**	Putative flavin amine oxidase	-	02670	3.9
**197**	Sulfide:quinone oxidoreductase	*sqr*	02710	4.4
**59**	Putative Zn metallo- β lactamase/putative rhodanese domain-containing protein	-	02720	6.9
**65**	Putative Zn metallo- β lactamase/putative rhodanese domain-containing protein	-	02720	11.4
**94**	Putative Zn metallo- β lactamase/putative rhodanese domain-containing protein	-	02720	3.9
**162**	Putative Zn metallo- β lactamase/putative rhodanese domain-containing protein	-	02720	7.4
**213**	Putative rhodanese-related sulfurtransferase	-	02740	11.0
**174**	Putative luciferase-like monooxygenase	-	05640	3.0
**9**	LSU ribosomal protein L10P	*rplJ*	09300	4.6
**191**	Putative alkyl hydroxperoxide reductase	-	13640	4.3
**64**	Putative quinoprotein amine dehydrogenase domain-containing protein	-	16720	3.1
**73**	Putrescine oxidase	*puo*	25690	5.8
**5**	Putative peptidyl-propyl-cis-trans isomerase binding protein	-	27670	5.1
**56**	Putative aldehyde dehydrogenase	-	54650	3.4
**171**	Pyridoxal 5´-phosphate synthase	*pdxS*	55960	3.9
**164**	Ketol-acid reductoisomerase (EC 1.1.1.86)	*ilvC*	63600	12.0
**85**	Putative acyl-CoA dehydrogenase	-	66330	4.4
**96**	Putative acyl-CoA dehydrogenase	-	66330	14.0
**100**	Putative acyl-CoA dehydrogenase	-	66330	12.3
**103**	Putative acyl-CoA dehydrogenase	-	66330	13.1
**104**	Putative acyl-CoA dehydrogenase	-	66330	13.9
**106**	Putative acyl-CoA dehydrogenase	-	66330	5.4
**156**	Alpha/beta hydrolase domain-containing protein	-	66340	4.8
**159**	Alpha/beta hydrolase domain-containing protein	-	66340	4.2

**Table 3:** Proteins exhibiting significantly increased expression of ≥ 3 during stationary growth phase in cells of *R*. *erythropolis* strain MI2 cultivated with DTDB (D) in comparison to cells cultivated with succinate (S).

Spot	Protein identity	Gene	ORF (RERY xxxxx)	Ratio D/S
**113**	Putative acetamidase/formamidase	*amdA / fmdA*	01230	6.6
**208**	Putative OsmC-like protein	-	02650	3.5
**197**	Sulfide:quinone oxidoreductase	*sqr*	02710	7.3
**58**	Putative Zn metallo- β lactamase/putative rhodanese domain-containing protein	-	02720	5.0
**59**	Putative Zn metallo- β lactamase/putative rhodanese domain-containing protein	-	02720	9.9
**65**	Putative Zn metallo- β lactamase/putative rhodanese domain-containing protein	-	02720	12.7
**80**	Putative Zn metallo- β lactamase/putative rhodanese domain-containing protein	-	02720	4.4
**162**	Putative Zn metallo- β lactamase/putative rhodanese domain-containing protein	-	02720	11.7
**211**	Superoxide dismutase [Mn]	*sodA*	05600	4.8
**54**	Chaperonin 1	*groL1*	13450	4.1
**191**	Putative alkyl hydroxperoxide reductase	-	13640	3.3
**64**	Putative quinoprotein amine dehydrogenase domain-containing protein	-	16720	5.9
**73**	Putrescine oxidase	*puo*	25690	3.6
**101**	Putrescine oxidase	*puo*	25690	13.5
**42**	Phosphoribosylaminoimidazolecarboxamide formyltransferase	*purH*	25830	3.4
**149**	Putative Zn-type alcohol dehydrogenase	-	50490	5.6
**56**	Putative aldehyde dehydrogenase	-	54650	4.3
**161**	Exodeoxyribonuclease III	*xthA*	57500	4.6
**61**	D-3-phosphoglycerate dehydrogenase	*serA*	63610	3.7
**164**	Ketol-acid reductoisomerase (EC 1.1.1.86)	*ilvC*	63600	5.2
**85**	Putative acyl-CoA dehydrogenase	-	66330	5.7
**100**	Putative acyl-CoA dehydrogenase	-	66330	4.2
**103**	Putative acyl-CoA dehydrogenase	-	66330	6.4
**104**	Putative acyl-CoA dehydrogenase	-	66330	7.3
**106**	Putative acyl-CoA dehydrogenase	-	66330	3.1
**117**	Putative acyl-CoA dehydrogenase	-	66330	8.5
**156**	Alpha/beta hydrolase domain-containing protein	-	66340	4.0

### Import of DTDB

The disulfide DTDB has to be imported into the cells of *R*. *erythropolis* MI2. This occurs probably *via* an active transporter system, as passive or facilitated diffusion is not feasible for a dicarboxylate. The conditions applied for disruption of the cells and protein extraction were not optimal for detection of membrane-associated proteins. However, some proteins involved in amino acid transport and two other transporter components could be identified (S2, S3 and S4 Tables), which displayed a slightly increased expression level (DTDB/Succ). These two proteins represent ATPase components of the ABC transporter family. The protein encoded by the gene with the locus tag RERY_53750 showed an expression level of 2.4 in the exponential phase and the other (RERY_39460) was expressed by a ratio of 1.9 in the stationary phase. The genome of *R*. *erythropolis* strain MI2 harbours 137 putative genes coding for proteins with an ATP-binding domain of ABC transporters. As mentioned in several other publications, the import of many toxic and xenobiotic compounds in *Rhodococci* was achieved by an active transport machinery. However, there are only few studies clearly identifying the mechanisms of transport in *Rhodococcus* strains [7] [49–52]. For example (I) the selective transport of n-hexadecane by *R*. *erythropolis* S+14He [53]; this strain has the ability to recognize the n-hexadecane from mixtures of similar molecules and transport it selectively into the cell by an energy driven mechanism. (II) The uptake of phthalates by *R*. *jostii* RHA1; cells of RHA1 could transport this compound *via* an active influx ABC transporter encoded by *patDABC* [54]. (III) The active transport of 4,6-dimethyldibenzothiophene (DBT) in *R*. *erythropolis* LSSE8-1. The rate of desulfurization of DBT increased after the effective transformation of the plasmid encoding the DBT uptake genes from *Pseudomonas delafieldii* R-8 into the wild type strain LSSE8-1 [55]. Accordingly, the two ABC transporter components (RERY_53750 and RERY_39460) identified in this study could be involved in the transport of DTDB into the cell, but this is speculative and requires further experiments.

### Cleavage of DTDB

The initial enzymatic reaction with DTDB as the substrate is performed by an NADH:flavin oxidoreductase Nox_MI2_ (RERY_03780). This reaction was confirmed in our previous studies [10, 19], which verified that DTDB is cleaved symmetrically into two molecules of 4MB in the presence of NADH by Nox_MI2_.

### Oxygenation of 4MB

**Fig 4:**
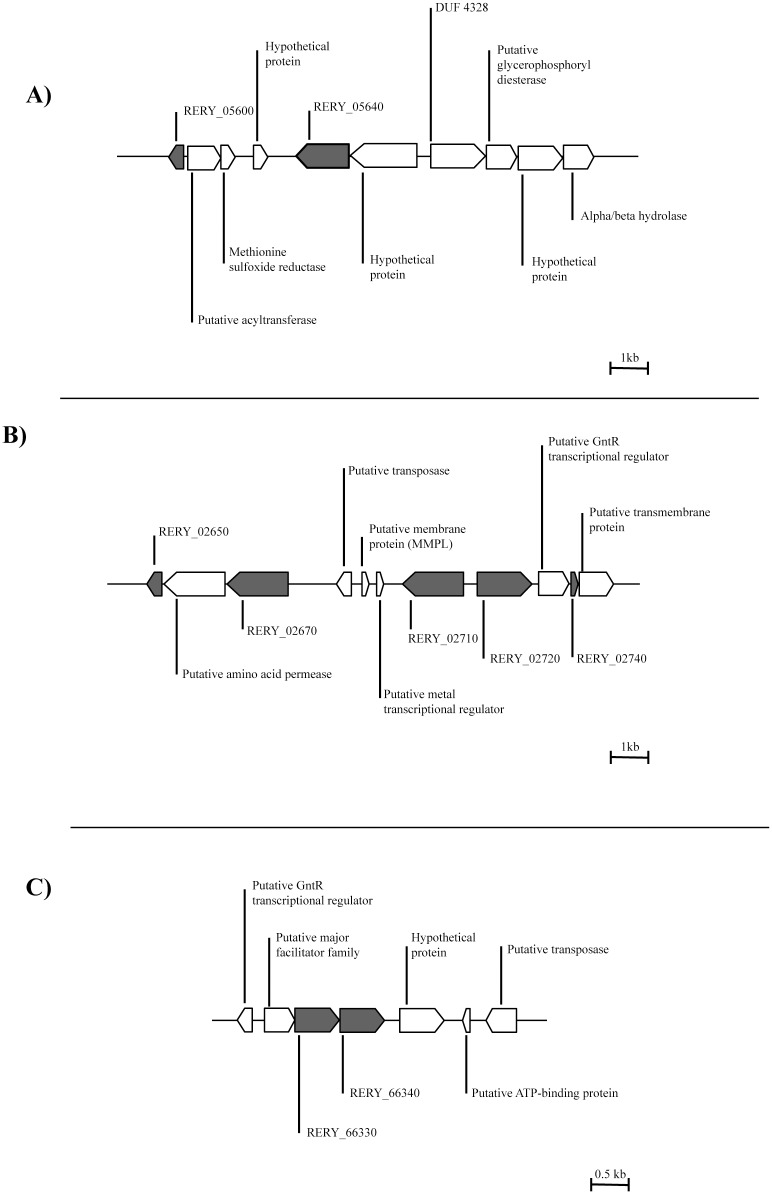
Organization of genes putatively involved in the degradation of DTDB. Genes encoding the remarkable proteins identified during proteome analysis are marked in gray. **A)** Cluster coding for (locus tags left to right): RERY_05600, superoxide dismutase; RERY_05640, putative luciferase-like monooxygenase F_420_-dependent enzyme. **B)** Cluster coding for (locus tags from left to right): RERY_02650, putative OsmC-like protein; RERY_02670, putative flavin amine oxidase; RERY_02710, putative sulfide: quinone oxidoreductase; RERY_02720, putative Zn metallo-beta lactamase/putative rhodanese domain-containing protein; RERY_02740, putative rhodanese-related sulfurtransferase. **C)** Cluster coding for (locus tags left to right): RERY_66330, putative acyl-CoA dehydrogenase; RERY_66340, alpha/beta hydrolase domain-containing protein.

**Fig 5:**
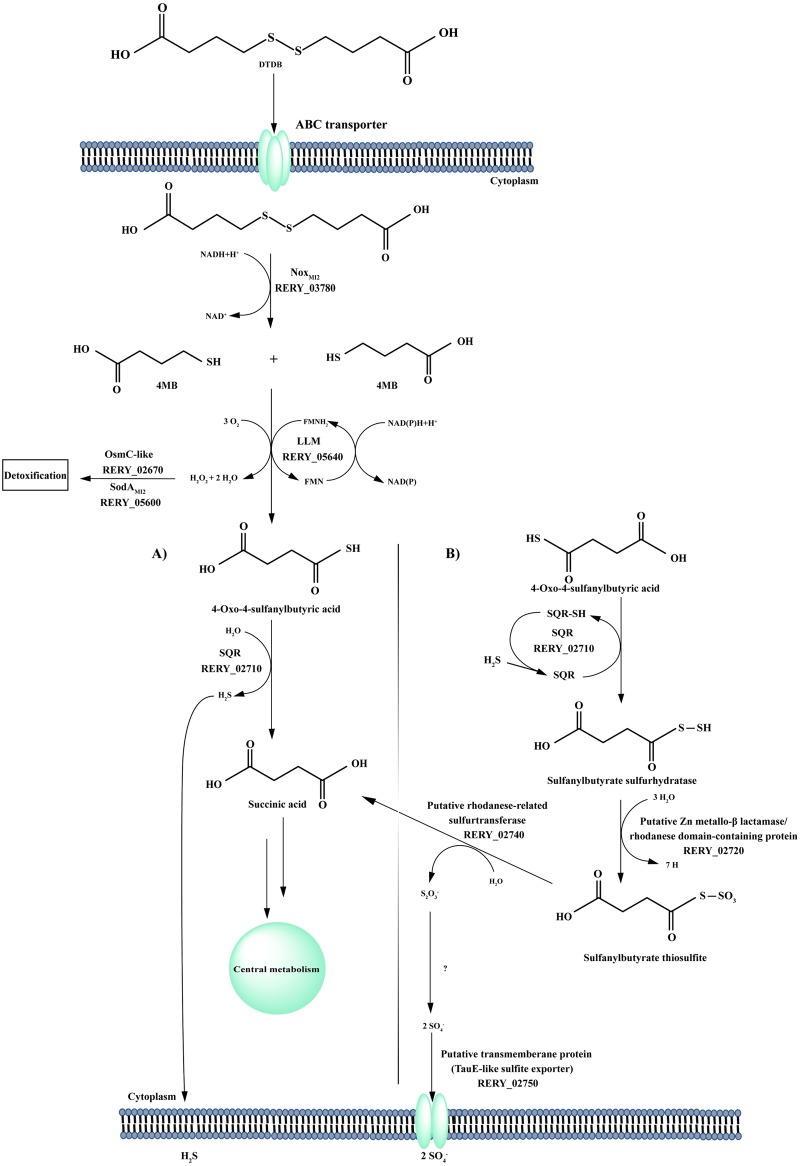
Updated degradation pathway of DTDB in *R*. *erythropolis* MI2. Nox, using NADH, cleaves DTDB into two molecules 4MB. Then, a putative oxygenase converts 4MB into 4-oxo-4-sulfanylbutyric acid. **A)** Desulfurization of 4-oxo-4-sulfanylbutyric acid by either the action of SQR or the putative desulfhydrase. **B)** Hypothesized alternative sulfur oxidation process. Abbreviations: Nox_MI2_, NADH:flavin oxidoreductase, LLM, luciferase-like monooxygenase; SQR, sulfide:quinone oxidoreductase; OsmC-like, osmotically induced protein; SodA_MI2_, superoxide dismutase.

A significant expression was detected for a protein encoded by the gene with the locus tag RERY_05640 (genomic region I, Fig 2), which could be annotated as a putative luciferase-like monooxygenase (LLM_MI2_) F_420_-dependent oxidoreductase. This protein showed a 2.8-fold increased expression level in the exponential growth phase (Fig 4A, Table 2). LLM are members of flavin-dependent monooxygenases. Thus, they usually have the ability to transfer molecular oxygen *via* a reduced flavin cofactor and incorporate one oxygen atom into the substrate of the corresponding enzyme [56, 57]. All family members catalyze a variety of chemical reactions and the common factor is the need of oxygen [58]. In 2010, comparative genomic approaches revealed that LLM can be associated with the F_420_-dependent coenzyme in *Mycobacteria tuberculosis* and other related mycobacteria and actinomycetes [59]. This enzyme from *M*. *tuberculosis* (accession number AY906857.1) showed a ketoreductase activity during the biosynthesis of mycobacterial virulence factors [60, 61]. In the genome of *R*. *erythropolis* MI2, 45 putative LLM-encoding genes were detected, but only the above mentioned LLM_MI2_ (RERY_05640,) exhibited a significant high expression ratio. Presumably, the second step in DTDB degradation is the mono-oxygenation of 4MB resulting in 4-oxo-4-sulfanylbutyric acid (Fig 1) and we postulate the participation of the gene with the locus tag RERY_05640 in the catabolic pathway of DTDB. A 4MB molecule is supposedly oxidized in the presence of reduced FMN, forming the 4-oxo-4-sulfanylbutyric acid and H_2_O as the products of this reaction (Fig 5).

### Removal of the sulfur group

The desulfhydration reaction of 4-oxo-4-sulfanylbutyric acid is still speculative. It is also very interesting as such type of enzymatic reaction would be completely novel. According to *in silico* analyses, only one gene in the MI2-genome could be annotated as a desulfhydrase (RERY_06500). This desulfhydrase shows a domain that affiliates it to the pyridoxal phosphate-dependent (PLP) enzymes, more specific to the D-cysteine desulfhydrase family (TIGR01275). PLP enzymes are described to act on D-amino acids, so they usually require an amino group to perform their reaction [62], but an amino group is apparently not present in 4-oxo-4-sulfanylbutyrate. This desulfhydrase is highly conserved in many strains of the genus *Rhodococcu*s, but an orthologue to RERY_06500 is completely missing in *R*. *jostii* RHA1. As described in a previous publication, *R*. *jostii* RHA1 was unable to grow in the presence of DTDB, even in the absence of other utilizable carbon sources [10]. *In silico* analyses showed that the genome of strain RHA1 harbours an orthologue of *nox* (ABG95452) and also the LLM F_420_-dependent oxidoreductase (ABG95816), whereas an orthologue to the putative desulfhydrase is absent. Hypothetically, strain RHA1 is able to import and cleave DTDB, yielding toxic 4MB and converting it to the presumably also highly toxic 4-oxo-4-sulfanylbutyrate. The latter would then accumulate in the cells because of the absence of a desulfhydrase and growth ceases. This strongly supports the importance of an operable desulfhydrase (RERY_06500) in the catabolism of DTDB. The next experimental steps will be a deletion of the desulfhydrase-encoding gene and later on enzyme assay studies, verifying the postulated reaction.

Another hypothesis for an alternative pathway of the sulfur-removal is based on the strong expression especially of three proteins encoded by the same cluster: (I) a sulfide: quinone oxidoreductase (SQR_MI2_) (RERY_02710) (II) a rhodanese-related sulfurtransferase (RERY_02740) and (III) a fused protein consisting of two large domains, a putative zinc metallo-beta lactamase domain and a rhodanese-like domain, encoded by the gene with the locus tag RERY_02720 (Tables 2 and 3) (Figs 4B and 5).

(I) Sulfide oxidation process is achieved by two known enzymatic systems: (i) flavocytochrome c and (ii) SQR [63]. In archaea and bacteria, H_2_S acts as an electron donor for the respiratory chain. Genes coding for SQR are well-described to be present in mitochondrial genomes, as well as in the human genome [64]. In this study, the SQR_MI2_ (RERY_02710) was identified as one of the highly expressed proteins; this enzyme is a member of the flavin disulfide reductase family, which includes dihydrolipoamide dehydrogenases, glutathione and thioredoxin reductases. Additionally, SQR catalyzes sulfide oxidation during sulfide-dependent chemo- and phototrophic growth in bacteria [65]. SQR donates electrons from sulfide to the electron transport chain at the level of quinone [66], e.g., the green sulfur bacterium *Chlorobaculum tepidum* has the ability to oxidize sulfide, elemental sulfur, and thiosulfate for photosynthetic growth applying its SQR [67]. Accordingly, it is possible in the DTDB catabolism that the enzyme encoded by RERY_02710 could perform either the desulfhydration process of 4-oxo-4-sulfanylbutyric acid, or the oxidation of the hydrogen sulfide formed after the desulfhydration of 4-oxo-4-sulfanylbutyric acid (Fig 5A and 5B).

(II) Rhodanese-related sulfurtransferases catalyze the transfer of a sulfur atom from a donor to a nucleophilic acceptor *in vitro*, for example from thiosulfate to cyanide [68]; however, donors and acceptors *in vivo* are difficult to identify [69]. The bovine liver rhodanese is the best-characterized sulfurtransferase, which has been the subject of numerous functional investigations [70–73]. Its mode of action was achieved *via* a double displacement mechanism involving the temporary formation of a persulfide-containing intermediate (Rhod-S), in which sulfur is bound to catalytic Cys residue [68]. Furthermore, oxygenase reactions catalyzed by *Rhodococcus* strains facilitate the growth and degradation of a wide range of pollutants and many *Rhodococcus* strains are known to perform such kind of reactions: sulfoxidation of sulfide to sulfoxide and subsequently sulfoxidation of sulfoxide to sulfone [3].

(III) Proteins with a metallo-beta lactamase domain are members of the beta-lactamase class, which includes thiolesterases, members of the glyoxalase II family [74]. Proteins of this class catalyze the hydrolysis of S-D-lactoyl-glutathione to form glutathione and D-lactic acid [74]. It was reported that zinc-type metallo-beta lactamases are responsible for the antibiotic resistance of a large number of pathogenic bacterial strains to penicillins and related antibiotics [74]. The Zn metallo-beta lactamase and the sulfur oxidizing (Sox) protein are belonging to the sulfur oxidase family, which is associated with sulfate metabolism [75]. This domain could be involved in the sulfur oxidation process in the DTDB catabolism. The second domain is a putative rhodanese-like domain. It was reported that some rhodanese domain-containing proteins are combined with other domains forming a “new” protein [68, 76]. This type of hybrid proteins appeared to be involved in the transport of sulfate [77] or in the metabolism of sulfur-containing molecules such as thiamin or molybdopterin [78]. Thus, we assume that the hybrid protein encoded by the gene with the locus tag RERY_02720 could play a vital role in DTDB degradation pathway (Fig 5). Consequently, this complete cluster (Fig 4B, genomic region II [Fig 2]) plays an important role in sulfur oxidation and transfer during the DTDB catabolism. A putative sulfite exporter (RERY_02750) could then supposedly export the sulfite/sulfate formed as a final product (Fig 5).

### Stress caused by DTDB degradation intermediates

Interestingly, a superoxide dismutase (SodA_MI2_, RERY_05600, genomic region I [Fig 2]), which is localized in direct proximity to the gene encoding the LLM F_420_-dependent oxidoreductase (RERY_05640; compare Fig 4A), showed a 4.8-fold increased spot volume (Table 3). The high expression of SodA_MI2_, which is usually involved in the detoxification of reactive oxygen species, indicates the increased stress level in cells utilizing DTDB. This is derived probably either due to the inevitably occurring degradation intermediates 4MB and 4-oxo-4-sulfanylbutyric acid or the sulfite/sulfide molecules formed.

Oxidative stress in cells is frequently caused by disulfides and thiols. Many reactive oxygen species (ROS), such as the superoxide anion and hydrogen peroxide (H_2_O_2_) are formed during the degradation or the formation of disulfides. Thiols are rapidly oxidized producing highly toxic peroxy compounds under aerobic conditions and in presence of heavy metals, which occur in several biochemical processes [79]. The utilization of DTDB as sole carbon source is apparently causing oxidative stress in the cells, which might result in DNA mutations or disturbed protein folding due to the increased level of ROS in the cell [80–82]. Several stress response-related proteins in addition to SodA_MI2_ were identified during proteome analysis, especially an OsmC-like protein (RERY_02650) and a putative alkyl hydroperoxide reductase (RERY_13640). The former was found to be strongly expressed in DTDB-grown cells during both exponential and stationary phases by a factor of 7.1-fold and 4.2-fold, respectively (Tables 2 and 3). OsmC-like proteins and organic hydroperoxide resistance proteins (Ohr) were identified as new members of the peroxidase family showing similar structures and functions to peroxidases [83, 84]. Initially, these proteins were known to be part of the bacterial response to osmotic stress [85]. After several biochemical studies, Ohr/OsmC proteins were characterized as cysteine-based, thiol-dependent peroxidases that reveal an ability to detoxify organic hydroperoxides [84, 86]. The catalytic mechanism of Ohr/Osmc is the oxidation of a cysteine by a hydroperoxide, thereby forming a sulfenic acid intermediate (-SOH) and then reducing the hydroperoxide to a corresponding alcohol. Subsequently, the sulfenic acid intermediate forms a disulfide bond with the second cysteine [83, 84, 87]. Interestingly, an orthologue of the putative OsmC-like protein (RERY_02650) could not be detected in the genomes of all other available *R*. *erythropolis* strains. Its amino acid sequence is 45% identical with the OsmC-like protein of *Actinomyes urogenitalis* and 41% with the OsmC-like protein of *R*. *opacus* B4. As 4MB is a highly toxic thiol, the expression of potent oxidative stress proteins are mandatory in cells of strain MI2 to survive the utilization of the xenobiotic DTDB as carbon source for growth.

### Other high abundant proteins

In addition to proteins involved more or less obviously in the DTDB degradation pathway, various other proteins with high expression levels became conspicuous during this study. The first is a putative acyl-CoA dehydrogenase (RERY_66330, genomic region III [Fig 2]), which was significantly stronger expressed in the exponential and the stationary phase (Tables 2 and 3) of DTDB-grown cells. Eleven isoforms of this protein were detected on the 2D-PAGE gels with an expression ratio of up to 14.0 compared to gels of succinate-grown cells (Fig 4C). *In silico* analysis revealed that this protein has no orthologues in genomes of other *R*. *erythropolis* strains. The second is an alpha/beta hydrolase domain-containing protein (RERY_66340), encoded by a gene localized upstream of the gene coding for the acyl-CoA dehydrogenase (Fig 4C). This protein was also highly expressed in the exponential and the stationary phase with an expression ratio up to 4.8 and 4.0 (DTDB/succ), respectively (Tables 2 and 3). Further investigations are required to clarify the role of these MI2-specific proteins during growth on DTDB.

Moreover, four isoforms of a putative flavin amine oxidase (RERY_02670) were identified showing an elevated expression level especially in the exponential phase with a ratio of 5.6 (DTDB/Succ) (Tables 2 and 3). Flavin amine oxidases have been identified in a variety of microorganisms including bacteria and fungi [88]. The main biological role of amine oxidases in lower eukaryotes and in bacteria is to provide the organisms with ammonium. Especially conspicuous is the upstream and downstream region of this oxidase, since the aforementioned OsmC-like protein (RERY_02650) and SQR_MI2_ (RERY_02710) are in direct proximity (Fig 4B, genomic region II [Fig 2]).

## Conclusions

Taking into account all presented results of the current study, an updated degradation pathway of DTDB is postulated (Fig 5). The transport system is still uncertain, but DTDB is initially cleaved by the action of Nox (RERY_03780) giving 4MB, which is then oxidized forming 4-oxo-4-sulfanylbutyric acid *via* the LLM_MI2_ F_420_-dependent enzyme (RERY_05640). The final step in the DTDB catabolism is the desulfurization, which could either be achieved by SQR_MI2_ (RERY_02710) or the putative desulfhydrase (RERY_06500) (Fig 5A, genomic region I [Fig 2]). H_2_S formed beside succinate by the reaction of the desulfhydrase could be oxidized by SQR and then reacts with a free thiol group forming a sulfurhydrate-containing compound. This compound may be oxidized forming thiosulfite by the fused protein encoded by RERY_02720, which is then subjected for further oxidation by the putative rhodanese-related sulfurtransferase (RERY_02740), giving sulfite and succinic acid as final products. Sulfite could be oxidized to sulfate before being exported outside the cell *via* the transmembrane exporter protein (RERY_02750), while succinic acid is most probably in any case utilized for growth (Fig 5B). Because of the possible formation of either ROS, H_2_O_2_ or a superoxide anion, the SodA_MI2_ (RERY_05600), the OsmC-like protein (RERY_02650) and the putative alkyl hydroperoxide reductase (RERY_13640) are presumably important for cell protection. The further biochemical analyses and mutation studies required to verify our postulations are ongoing and will be completed in the future.

## Supporting Information

S1 Fig Pulsed-field gel (PFG) profiles of extrachromosomal DNA from strain MI2. Approximately 2 μg of the lambda ladder PFG marker were loaded to determine the migrating size of the megaplasmids. Chromosomal (chrom.) and distinguishable species of plasmids, as well as relevant ladder sizes (in kb) are labeled. Each lane represents either one or two 80 μl plugs containing 100 μg cells/μl: lane 1, two plugs; lane 2, one plug.
(TIF)

S2 Fig Growth of *R*. *erythropolis* MI2 incubated with DTDB (♦) or succinate (▲) as sole source of carbon. The optical density was determined using a Klett-Summerson photometer. **(˟)** Concentration of DTDB during the growth of strain MI2 for proteome analysis. Measurement was performed by GC/MS analysis of the lyophilized supernatant.
(TIF)

S3 Fig SDS-PAGE illustrating the successful extraction of proteins from cells of *R*. *erythropolis* MI2 cultivated with DTDB (D) or succinate (S). (TIF)

S4 Fig Exponential phase: Dual channel image generated by the software Delta2D 4.2. The image showing the difference in the proteome of *R*. *erythropolis* MI2 cultivated with DTDB (orange spots) or succinate (blue spots) as revealed by 2D-PAGE. Black spots represent equally expressed proteins. Blue spots indicate proteins with decreased expression of ≤ 0.5 during growth with DTDB while orange spots indicate proteins with increased expression of ≥ 2 during growth with DTDB. Labelled spots were successfully identified by MALDI-TOF-MS/MS.
(TIF)

S5 Fig Stationary phase: Dual channel image generated by the software Delta2D 4.2. The image illustrating the difference in the proteome of *R*. *erythropolis* MI2 cultivated with DTDB (orange spots) or succinate (blue spots) as revealed by 2D-PAGE. Black spots represent equally expressed proteins. Blue spots indicate proteins with decreased expression of ≤ 0.5 during growth with DTDB while orange spots indicate proteins with increased expression of ≥ 2 during growth with DTDB. Labelled spots were successfully identified by MALDI-TOF-MS/MS.
(TIF)

S1 Table Exponential growth phase: Proteins exhibiting significantly increased expression of ≥ 2 in cells of *R*. *erythropolis* strain MI2 cultivated with DTDB (D) in comparison to cells cultivated with succinate (S). (PDF)

S2 Table Proteins exhibiting an expression level of ≤ 2 during exponential growth phase in cells of *R*. *erythropolis* strain MI2 cultivated with DTDB (D) in comparison to cells cultivated with succinate (S). (PDF)

S3 Table Stationary growth phase: Proteins exhibiting significantly increased expression of ≥ 2 in cells of *R*. *erythropolis* strain MI2 cultivated with DTDB (D) in comparison to cells cultivated with succinate (S). (PDF)

S4 Table Proteins exhibiting an expression level of ≤ 2 during the stationary growth phase in cells of *R*. *erythropolis* strain MI2 cultivated with DTDB (D) in comparison to cells cultivated with succinate (S). (PDF)

S5 Table Excel file with the spot volumes of all gels. (XLSX)

## Abbreviations

DTDB: 4,4´-dithiodibutyric acid
DTDP: 3,3´-dithiodipropionic acid
MALDI-TOF: matrix-assisted laser desorption ionization-time of flight tandem
4MB: 4-mercaptobutyric acid
NB: nutrient broth
OSC: organic sulfur compound
PTE: polythioester
