# Supplementary figures and images for: Genome and Proteome Analysis of Rhodococcus erythropolis MI2: Elucidation of the 4,4´-Dithiodibutyric Acid Catabolism
Source: PLoS One. 2016 Dec 15;11(12):e0167539. doi: 10.1371/journal.pone.0167539 (PMC5157978; doi:10.1371/journal.pone.0167539)

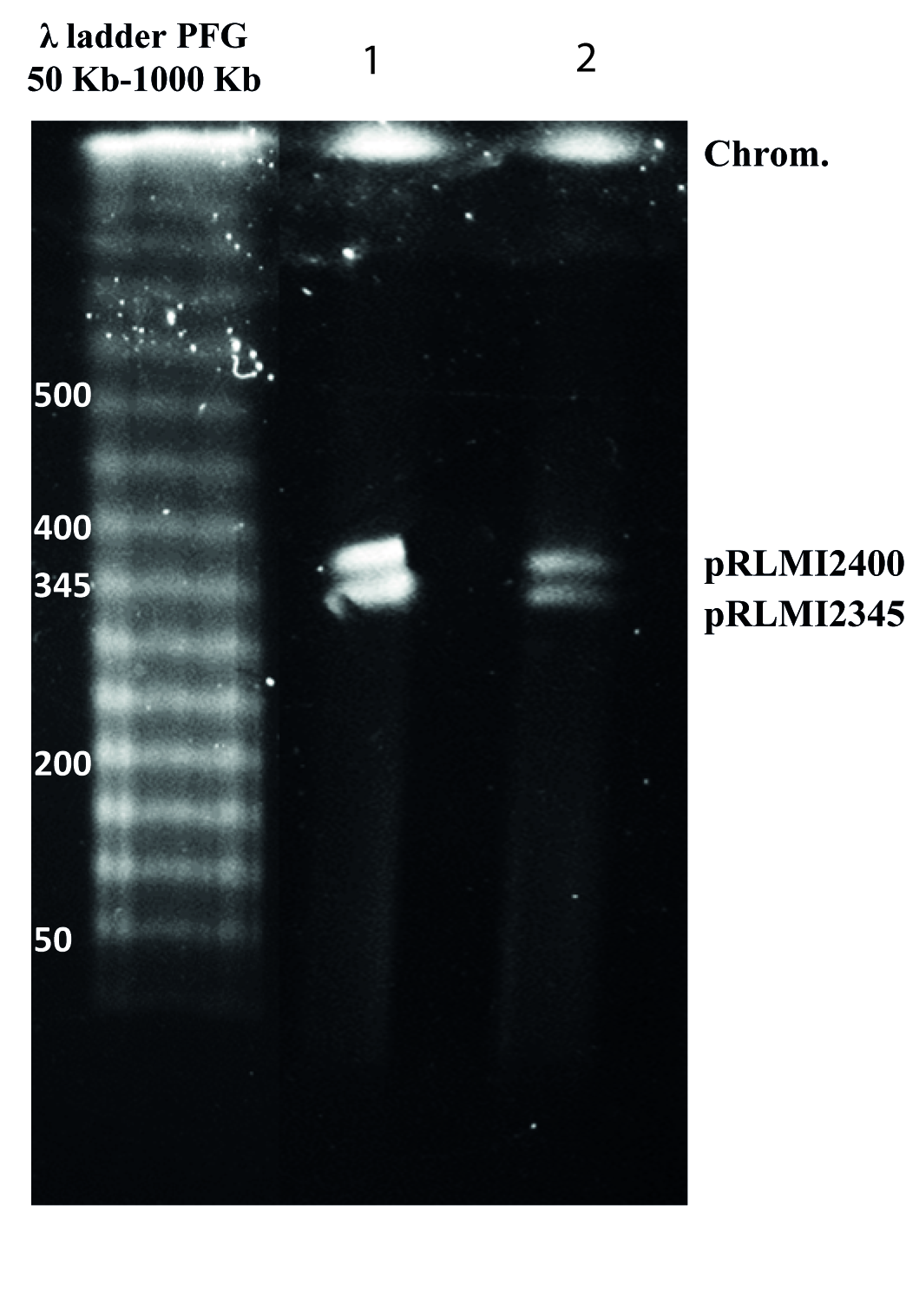

Supplement: S1 Fig — Approximately 2 μg of the lambda ladder PFG marker were loaded to determine the migrating size of the megaplasmids. Chromosomal (chrom.) and distinguishable species of plasmids, as well as relevant ladder sizes (in kb) are labeled. Each lane represents either one or two 80 μl plugs containing 100 μg cells/μl: lane 1, two plugs; lane 2, one plug. (TIF) [file pone.0167539.s001.tif]

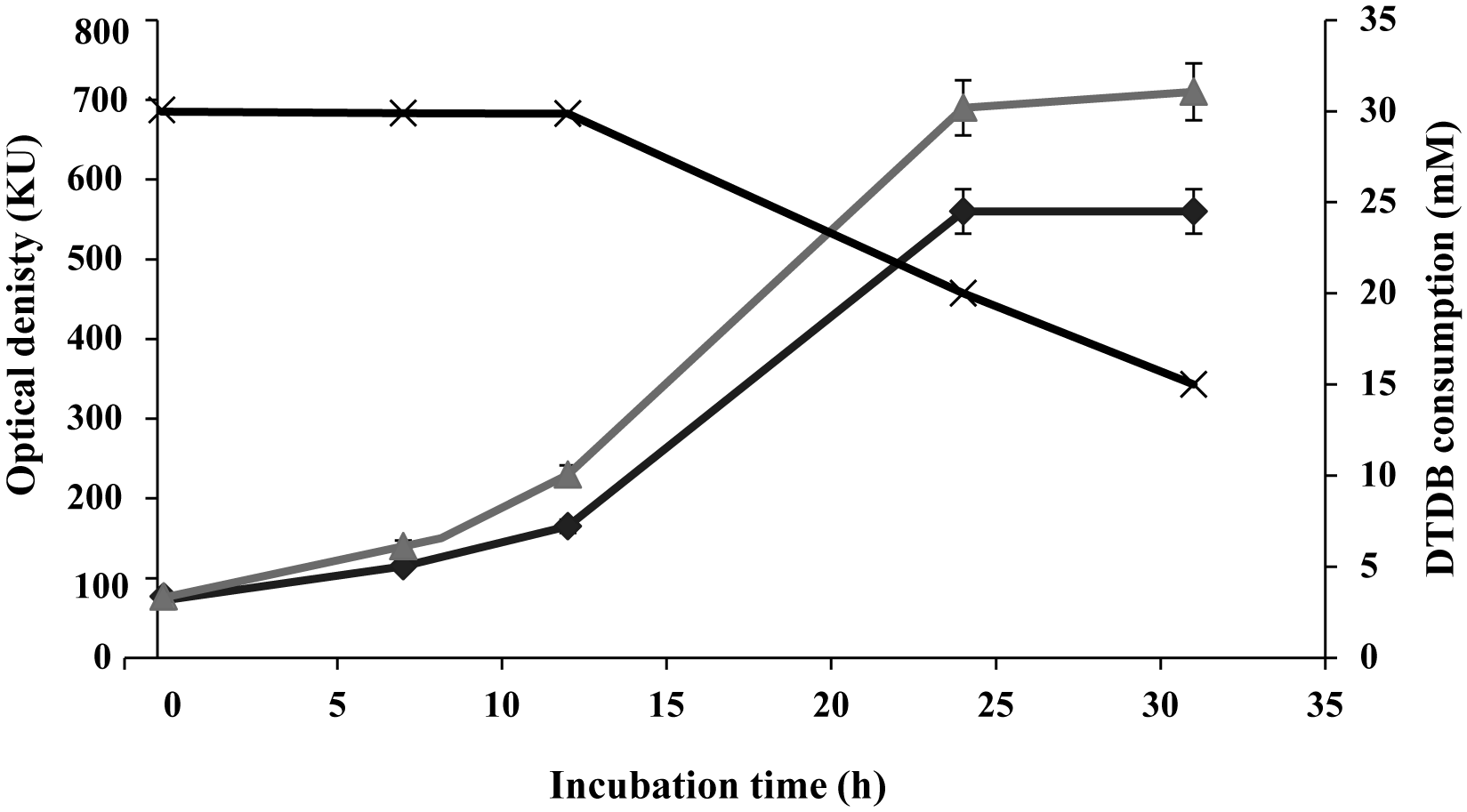

Supplement: S2 Fig — The optical density was determined using a Klett-Summerson photometer. (˟) Concentration of DTDB during the growth of strain MI2 for proteome analysis. Measurement was performed by GC/MS analysis of the lyophilized supernatant. (TIF) [file pone.0167539.s002.tif]

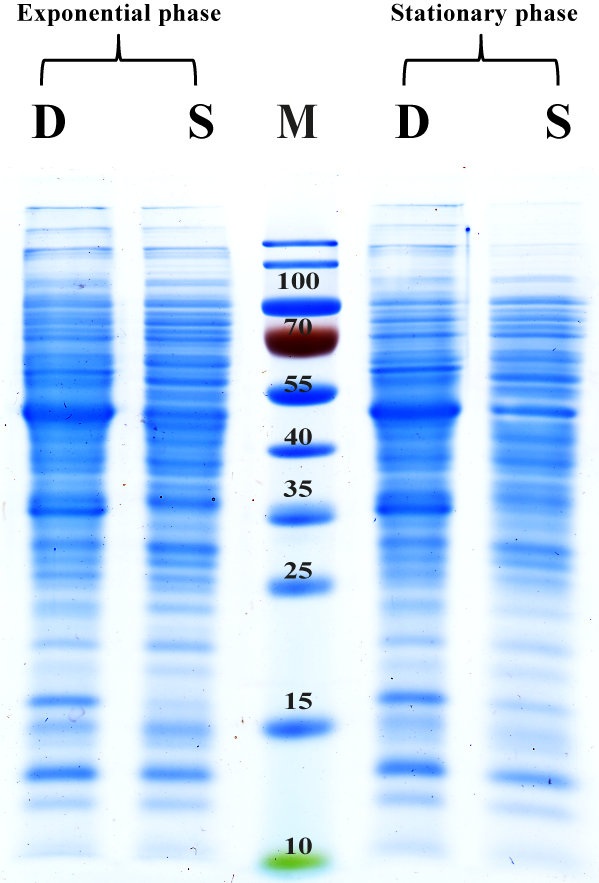

Supplement: S3 Fig — (TIF) [file pone.0167539.s003.tif]

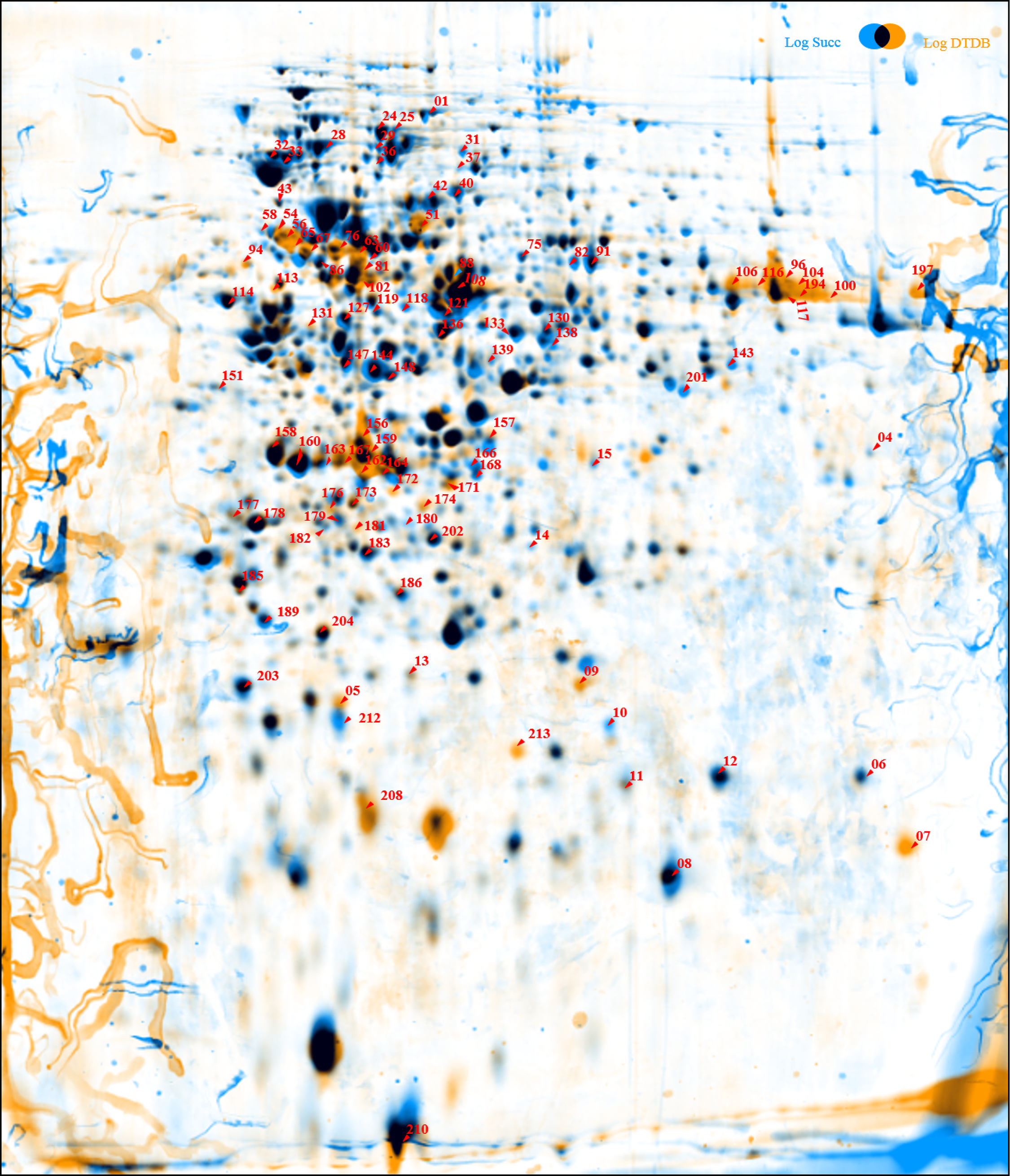

Supplement: S4 Fig — The image showing the difference in the proteome of R. erythropolis MI2 cultivated with DTDB (orange spots) or succinate (blue spots) as revealed by 2D-PAGE. Black spots represent equally expressed proteins. Blue spots indicate proteins with decreased expression of ≤ 0.5 during growth with DTDB while orange spots indicate proteins with increased expression of ≥ 2 during growth with DTDB. Labelled spots were successfully identified by MALDI-TOF-MS/MS. (TIF) [file pone.0167539.s004.tif]

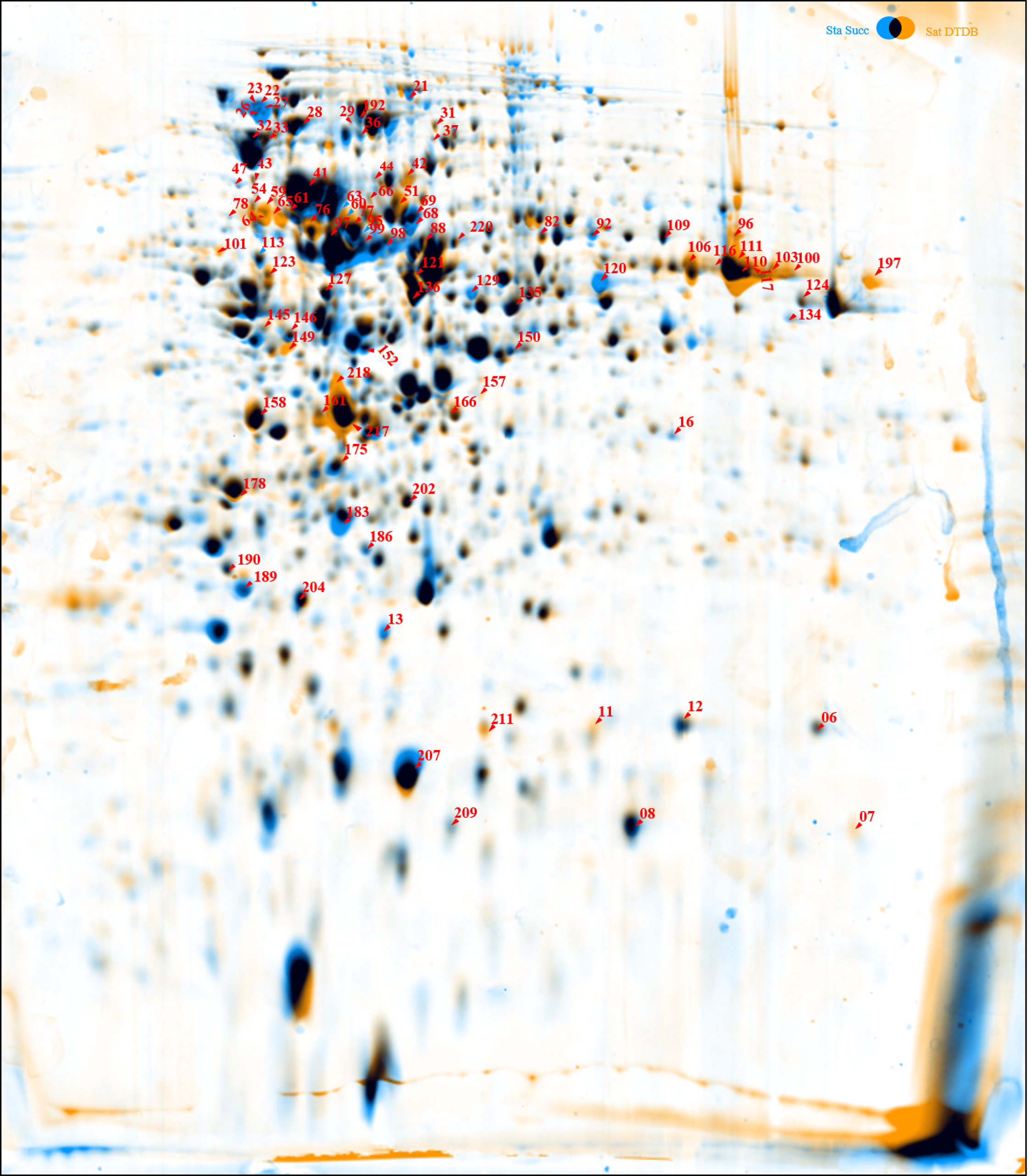

Supplement: S5 Fig — The image illustrating the difference in the proteome of R. erythropolis MI2 cultivated with DTDB (orange spots) or succinate (blue spots) as revealed by 2D-PAGE. Black spots represent equally expressed proteins. Blue spots indicate proteins with decreased expression of ≤ 0.5 during growth with DTDB while orange spots indicate proteins with increased expression of ≥ 2 during growth with DTDB. Labelled spots were successfully identified by MALDI-TOF-MS/MS. (TIF) [file pone.0167539.s005.tif]
