# Supplementary material for: Genome and Proteome Analysis of Rhodococcus erythropolis MI2: Elucidation of the 4,4´-Dithiodibutyric Acid Catabolism
Source: PLoS One. 2016 Dec 15;11(12):e0167539. doi: 10.1371/journal.pone.0167539 (PMC5157978; doi:10.1371/journal.pone.0167539)
Supplement: S1 Table — (PDF) [file pone.0167539.s006.pdf]

S1 Table.

Spot	Protein identity	Gene	ORF (RERY xxxxx)	Ratio D/S
5	Putative peptidyl-propyl-cis-trans isomerase binding protein	-	27670	5.1
9	LSU ribosomal protein L10P	*rplJ*	09300	4.6
37	Putative ABC transporter	-	53750	2.4
51	Gamma-aminobutyraldehyde dehydrogenase	*prr*	25710	2.5
56	Putative aldehyde dehydrogenase	-	54650	3.4
59	Putative Zn metallo-β lactamase/putative rhodanese domain-containing protein	-	02720	6.9
64	Putative quinoprotein amine dehydrogenase domain-containing domain	-	16720	3.1
65	Putative Zn metallo-β lactamase/putative rhodanese domain-containing protein	-	02720	11.4
73	Putrescine oxidase	*puo*	25690	5.8
76	D-3-phosphoglycerate dehydrogenase	*serA*	63610	2.0
80	Putative Zn metallo-β lactamase/putative rhodanese domain-containing protein	-	02720	2.6
81	Putative flavin amine oxidase	-	02670	5.6
85	Putative acyl-CoA dehydrogenase	-	66330	4.4
87	Hypothetical protein	-	04070	2.1
94	Putative Zn metallo-β lactamase/putative rhodanese domain-containing protein	-	02720	3.9
96	Putative acyl-CoA dehydrogenase	-	66330	14.0
97	Putative flavin amine oxidase	-	02670	2.0
99	Putative aminohydrolase	-	60890	2.1
100	Putative acyl-CoA dehydrogenase	-	66330	12.3
102	Putative flavin amine oxidase	-	02670	3.9
103	Putative acyl-CoA dehydrogenase	-	66330	13.1
104	Putative acyl-CoA dehydrogenase	-	66330	13.8
106	Putative acyl-CoA dehydrogenase	-	66330	5.4
109	Glutamate dehydrogenase	*gdhA*	53910	2.9

S1 Table Continued

Spot	Protein identity	Gene	ORF (RERY xxxxx)	Ratio D/S
110	Putative acyl-CoA dehydrogenase	-	66330	2.7
111	Putative acyl-CoA dehydrogenase	-	66330	8.0
113	Acetamidase/formamidase	*amdA/fmdA*	01230	5.5
117	Putative acyl-CoA dehydrogenase	-	66330	5.8
149	Putative alcohol dehydrogenase zinc type	-	50490	2.6
156	Alpha/beta hydrolase domain-containing protein	-	66340	4.8
159	Alpha/beta hydrolase domain-containing protein	-	66340	4.2
161	Exodeoxyribonuclease III	*xthA*	57500	2.4
162	Putative Zn metallo-β lactamase/putative rhodanese domain-containing protein	-	02720	7.4
164	Ketol-acid reductoisomerase (EC 1.1.1.86)	*ilvC*	63600	12.0
167	Translation elongation factor 1A (EF-1A/EF-Tu)	*tufA*	08860	2.6
171	Pyridoxal 5′-phosphate synthase	*pdxS*	55960	3.9
174	Putative luciferase-like monooxygenase	-	05640	2.8
176	Putative 3-hydroxybutyryl-CoA dehydrogenase	-	11770	2.1
181	Putative Zn metallo-β lactamase/putative rhodanese domain-containing protein	-	02720	2.2
191	Putative alkyl hydroxperoxide reductase	-	13640	4.3
194	Putative acyl-CoA dehydrogenase	-	66330	7.5
197	Sulfide:quinone oxidoreductase	*sqr*	02710	4.4
207	Putative OsmC-like protein	-	02650	7.1
208	Putative OsmC-like protein	-	02650	3.5
210	Putative cold-shock DNA-binding protein family	-	34650	2.3
213	Putative rhodanese-related sulfurtransferase	-	02740	11.0
