# Supplementary material for: Genome and Proteome Analysis of Rhodococcus erythropolis MI2: Elucidation of the 4,4´-Dithiodibutyric Acid Catabolism
Source: PLoS One. 2016 Dec 15;11(12):e0167539. doi: 10.1371/journal.pone.0167539 (PMC5157978; doi:10.1371/journal.pone.0167539)
Supplement: S2 Table — (PDF) [file pone.0167539.s007.pdf]

S2 Table.

Spot	Protein identity	Gene	ORF (RERY xxxxx)	Ratio D/S
1	ATP- depenent chaperone protein	*clpB*	23650	0.5
4	Putative formyltetrahydrofolate deformylase	-	01430	0.9
6	Protein of unknown function DUF520	-	09430	0.6
11	Transcriptional regulator, CarD family	*carD*	32860	0.6
12	Peptidyl-prolyl cis-trans isomerase A	*ppiA*	04110	0.5
13	ATP-dependent Clp protease proteolytic subunit 2	*clpP2*	53540	1.5
14	Enoyl-CoA- dehydratase	*echA*	08060	0.6
15	Putative aldolase class II	-	26090	1.1
16	Ribosomal protein 30S	*riaA*	61850	0.8
20	Catalase-peroxidase 2	*katG*	21510	0.8
21	ATP-dependent chaperone protein ClpB	*clpB1*	23650	1.2
22	Prolyl-tRNA synthetase (EC 6.1.1.15)	*proS*	65200	0.7
23	Catalase-peroxidase 2	-	21510	1.1
24	Malate synthase (EC 2.3.3.9)	-	44320	0.8
25	Translation elongation factor 2 (EF-2/EF-G)	*fusA*	08870	0.6
26	Catalase-peroxidase 2	-	21510	0.8
27	Methionine synthase (B12-independent)	*metE*	28700	0.4
28	Chaperone protein HtpG	*htpG*	48230	0.5
29	Phosphate acetyltransferase	*pta*	55200	0.4
31	Phosphate acetyltransferase	*pta*	55200	0.5
32	Chaperone protein DnaK	*dnaK*	23240	0.5
33	Phosphoenolpyruvate carboxykinase [GTP]	-	46200	0.6
36	Acetate--CoA ligase	*acsA*	34800	0.7
40	Biotin carboxylase (EC 6.3.4.14)	*accA*	61340	0.5
41	Chaperonin 1	*groL1*	13450	0.7

S2 Table Continued

Spot	Protein identity	Gene	ORF (RERY xxxxx)	Ratio D/S
42	Phosphoribosylaminoimidazolecarboxamide formyltransferase	*purH*	25830	0.6
43	Methylmalonyl-CoA mutase small subunit	*mutA*	39140	0.5
44	ATP synthase F1 subcomplex alpha subunit	*atpA*	31210	0.6
47	Phosphoenolpyruvate-protein phosphotransferase	-	16870	0.5
54	Chaperonin 1	*groL1*	13450	0.5
58	Putative Zn metallo-β lactamase/putative rhodanese domain-containing protein	-	02720	0.3
60	Putative acyl transferase domain-containing protein	-	54210	1.2
61	D-3-phosphoglycerate dehydrogenase	*serA*	63610	1.1
63	Putative ferredoxin reductase	-	54890	1.9
53	Putative deamidase	-	43890	0.4
66	Putative flavin amine oxidase	-	02670	1.5
67	D-3-phosphoglycerate dehydrogenase	*serA*	63610	1.3
68	3-isopropylmalate dehydratase large subunit	-	63710	0.8
69	Glutamine synthetase 1	*glnA1*	51630	1.2
75	Putative inosine-5'-monophosphate dehydrogenase	-	39040	0.6
77	Hypothetical protein	-	04070	1.7
78	Glycerol kinase (EC 2.7.1.30)	*glpK*	27610	1.7
82	3-deoxy-D-arabinoheptulosonate-7-phosphate synthase	-	51180	0.3
86	Putative aldehyde dehydrogenase	-	67160	0.9
88	Hypothetical protein	-	04070	1.5
91	Dihydrolipoamide dehydrogenase (EC 1.8.1.4)	*lpdA*	13270	0.4
92	dihydrolipoamide dehydrogenase (EC 1.8.1.4)	*lpdA*	13270	0.8
101	Putrescine oxidase	*puo*	25690	1.9
108	Isocitrate lyase (EC 4.1.3.1)	*aceA*	01250	1.4
114	Enolase	*eno*	24350	0.8
116	Putative acyl-CoA dehydrogenase	*acdA*	66330	1.8

S2 Table Continued

Spot	Protein identity	Gene	ORF (RERY xxxxx)	Ratio D/S
118	Putative methyltransferase	-	63250	0.3
119	Hypothetical protein	-	38010	0.7
120	Methanol dehydrogenase (acceptor) apoprotein (EC 1.1.99.37)	-	08730	1.0
121	Methionine adenosyltransferase (EC 2.5.1.6)	*metK*	40140	0.5
123	Fructose-1,6-bisphosphatase class 2	*glpX*	21280	0.8
124	Putative acyl-CoA dehydrogenase	-	06810	0.5
127	Putative acyl-CoA dehydrogenase	-	08290	0.8
129	Putative sorbitol dehydrogenase	-	30100	0.6
130	Beta-ketothiolase BktB	*bktB*	37770	0.7
131	Succinate--CoA ligase subunit beta	*sucC*	25890	1.4
133	Putative 3-ketoacyl-CoA thiolase	-	06740	0.5
134	Mycofactocin system (heme/flavin dehydrogenase)	*mftD*	08480	1.1
135	Beta-ketothiolase BktB	*bktB*	37770	0.3
138	Beta-ketothiolase BktB	*bktB*	37770	0.3
139	Quinolinate synthetase type A (EC 2.5.1.72)	-	50200	0.6
143	Putative luciferase-like monooxygenase	-	02920	0.5
144	NADPH-dependent curcumin reductase	*curA*	32210	0.5
145	Putative β-lactamase	-	10130	1.8
146	Acyl-CoA dehydrogenase	*acdA*	06760	1.0
147	Putative adenosine deaminase	-	60780	0.5
148	Aspartate-semialdehyde dehydrogenase	*asd*	35520	0.6
150	2,3,4,5-tetrahydropyridine-2,6-dicarboxylate N-succinyltransferase	*dapD*	56400	0.6
151	Thioredoxin reductase	*trxB*	03790	0.6
152	Aspartate-semialdehyde dehydrogenase	*asd*	35520	2.0
154	Aspartate-semialdehyde dehydrogenase	*asd*	35520	1.3

S2 Table Continued

Spot	Protein identity	Gene	ORF (RERY xxxxx)	Ratio D/S
157	Putative ATP-binding transporter	-	39460	0.6
158	Electron transfer flavoprotein alpha subunit apoprotein	*etfA*	63340	1.4
160	Putative thiosulfate sulfurtransferase (EC 2.8.1.1)	-	31860	0.8
163	Putative thiosulfate sulfurtransferase (EC 2.8.1.1)	-	31860	0.9
166	Citrate lyase subunit beta-like protein	-	64520	0.4
168	Putative fatty acid desaturase desA1	-	31650	0.3
169	Citrate lyase subunit beta-like protein	-	64520	0.6
173	Putative fumarylacetoacetate hydrolase domain-containing protein	-	63630	1.5
175	NAD(P) domain containing protein	-	56980	1.3
177	Electron transfer flavoprotein beta subunit	*etfB*	63330	2.0
178	Electron transfer flavoprotein beta subunit	*etfB*	63330	1.4
179	Putative encapsulating protein	-	03200	0.9
180	Putative phosphate transport system regulatory protein	-	31960	0.3
182	Putative thiazole synthase	-	55300	0.6
183	Electron transfer flavoprotein beta subunit	*etfB*	63330	0.9
185	Putative tellurium resistance protein	-	67580	1.6
186	NAD(P)-binding domain-containing protein	-	25340	1.1
189	Isochorismatase family protein	*yecD*	02630	0.3
190	Isochorismatase family protein	*yecD*	02630	1.4
192	Translation elongation factor 2 (EF-2/EF-G)	*fusA*	08870	0.4
201	Putative taurine catabolism dioxygenase	-	58710	0.2
202	Putative phosphoglycerate mutase	-	01700	0.8
204	ATP-dependent Clp protease, proteolytic subunit 2	*clpP*	53530	1.4
209	Putative pyridoxamine 5'-phosphate oxidase	-	57780	1.0
211	Superoxide dismutase [Mn]	*sodA*	05600	0.8
212	Putative peptidyl-propyl-cis-trans isomerase binding protein	-	27670	0.2
