# Supplementary material for: Genome and Proteome Analysis of Rhodococcus erythropolis MI2: Elucidation of the 4,4´-Dithiodibutyric Acid Catabolism
Source: PLoS One. 2016 Dec 15;11(12):e0167539. doi: 10.1371/journal.pone.0167539 (PMC5157978; doi:10.1371/journal.pone.0167539)
Supplement: S3 Table — (PDF) [file pone.0167539.s008.pdf]

S3 Table.

Spot	Protein identity	Gene	ORF (RERY xxxxx)	Ratio D/S
1	ATP- dependent chaperone protein	*clpB*	23650	2.5
4	Putative formyltetrahydrofolate deformylase	-	01430	2.1
11	Transcriptional regulator, CarD family	*carD*	32860	2.1
42	Phosphoribosylaminoimidazolecarboxamide formyltransferase	*purH*	25830	3.4
51	Gamma-aminobutyraldehyde dehydrogenase	*prr*	25710	2.7
54	Chaperonin 1	*groL1*	13450	4.1
56	Putative aldehyde dehydrogenase	-	54650	4.3
58	Putative Zn metallo-β lactamase/putative rhodanese domain-containing protein	-	02720	5.0
59	Putative Zn metallo-β lactamase/putative rhodanese domain-containing protein	-	02720	9.9
61	D-3-phosphoglycerate dehydrogenase	*serA*	63610	3.7
64	Putative quinoprotein amine dehydrogenase domain-containing protein	-	16720	5.9
65	Putative Zn metallo-β lactamase/putative rhodanese domain-containing protein	-	02720	12.7
73	Putrescine oxidase	*puo*	25690	3.6
76	D-3-phosphoglycerate dehydrogenase	*serA*	63610	2.3
80	Putative Zn metallo-β lactamase/putative rhodanese domain-containing protein	-	02720	4.4
85	Putative acyl-CoA dehydrogenase	-	66330	5.7
96	Putative acyl-CoA dehydrogenase	-	66330	2.6
100	Putative acyl-CoA dehydrogenase	-	66330	4.2
101	Putrescine oxidase	*puo*	25690	13.5
103	Putative acyl-CoA dehydrogenase	-	66330	6.4
104	Putative acyl-CoA dehydrogenase	-	66330	7.3
106	Putative acyl-CoA dehydrogenase	-	66330	3.1

S3 Table Continued

Spot	Protein identity	Gene	ORF (RERY xxxxx)	Ratio D/S
109	Glutamate dehydrogenase	*gdhA*	53910	2.6
111	Putative acyl-CoA dehydrogenase	-	66330	2.3
113	Acetamidase/formamidase	*amdA / fmdA*	01230	6.6
117	Putative acyl-CoA dehydrogenase	-	66330	8.5
149	Putative alcohol dehydrogenase zinc type	-	50490	5.6
151	Thioredoxin reductase	*trxB*	03790	2.7
156	Alpha/beta hydrolase domain-containing protein	-	66340	4.0
159	Alpha/beta hydrolase domain-containing protein	-	66340	2.8
161	Exodeoxyribonuclease III	*xthA*	57500	4.6
162	Putative Zn metallo-β lactamase/putative rhodanese domain-containing protein	-	02720	11.7
164	Ketol-acid reductoisomerase (EC 1.1.1.86)	*ilvC*	63600	5.2
168	Putative fatty acid desaturase	-	31650	2.3
191	Putative alkyl hydroxperoxide reductase	-	13640	3.3
194	Putative acyl-CoA dehydrogenase	-	66330	2.8
197	Sulfide:quinone oxidoreductase	*sqr*	02710	7.3
211	Superoxide dismutase [Mn]	*sodA*	05600	4.8
