# Supplementary material for: Genome and Proteome Analysis of Rhodococcus erythropolis MI2: Elucidation of the 4,4´-Dithiodibutyric Acid Catabolism
Source: PLoS One. 2016 Dec 15;11(12):e0167539. doi: 10.1371/journal.pone.0167539 (PMC5157978; doi:10.1371/journal.pone.0167539)
Supplement: S4 Table — (PDF) [file pone.0167539.s009.pdf]

S4 Table.

Spot	Protein identity	Gene	ORF (RERY xxxxx)	Ratio D/S
5	Putative peptidyl-propyl-cis-trans isomerase binding protein	-	27670	1.0
6	Protein of unknown function DUF520	-	09430	0.8
9	LSU ribosomal protein L10P	*rplJ*	-	2.0
10	Orotate phosphoribosyltransferase (EC 2.4.2.10)	*pyrE*	23690	0.8
12	Putative peptidyl-prolyl cis-trans isomerase A	-	04110	0.8
13	ATP-dependent Clp protease proteolytic subunit 2	*clpP2*	53540	0.3
14	Enoyl-CoA- dehydratase	*echA*	08060	0.5
15	Putative aldolase class II	-	26090	0.7
16	Ribosomal protein 30S	*riaA*	61850	0.3
20	Catalase-peroxidase 2	-	21510	0.2
21	ATP-dependent chaperone protein ClpB	*clpB*	23650	0.3
22	Prolyl-tRNA synthetase (EC 6.1.1.15)	*proS*	65200	0.2
23	Catalase-peroxidase 2	-	21510	0.3
24	Malate synthase (EC 2.3.3.9)	*glcB*	44320	1.3
25	Translation elongation factor 2 (EF-2/EF-G)	*fusA*	08870	1.5
26	Catalase-peroxidase 2	-	21510	0.2
27	Methionine synthase (B12-independent)	*metE*	28700	0.1
28	Chaperone protein HtpG	*htpG*	48230	0.6
29	Phosphate acetyltransferase	*pta*	55200	0.5
31	Phosphate acetyltransferase	*pta*	55200	1.3
32	Chaperone protein DnaK	*dnaK*	23240	0.9
33	Phosphoenolpyruvate carboxykinase [GTP]	-	46200	1.1
36	Acetate--CoA ligase	*acsA*	34800	0.4
37	Putative ABC transporter	-	53750	0.8

S4 Table Continued

Spot	Protein identity	Gene	ORF (RERY xxxxx)	Ratio D/S
40	Biotin carboxylase (EC 6.3.4.14)	*accA*	61340	1.0
41	Chaperonin 1	*groL1*	13450	1.1
43	Methylmalonyl-CoA mutase small subunit	*mutA*	39140	1.5
44	ATP synthase F1 subcomplex alpha subunit	*atpA*	31210	0.6
47	Phosphoenolpyruvate-protein phosphotransferase	-	16870	0.4
60	Acyl transferase domain-containing protein	-	54210	1.6
63	Putative ferredoxin reductase	-	54890	1.2
53	Putative deamidase	-	43890	1.2
66	Putative flavin amine oxidase	-	02670	1.9
67	D-3-phosphoglycerate dehydrogenase	*serA*	63610	1.0
68	3-isopropylmalate dehydratase large subunit	-	63710	0.3
69	Glutamine synthetase 1	*glnA1*	51630	0.4
75	Putative inosine-5'-monophosphate dehydrogenase	-	39040	0.9
77	Hypothetical protein	-	04070	0.8
78	Glycerol kinase (EC 2.7.1.30)	*glpK*	27610	0.3
81	Putative flavin amine oxidase	-	02670	1.2
82	3-deoxy-D-arabinoheptulosonate-7-phosphate synthase (EC 2.5.1.54)	*aroH*	51180	1.6
86	Putative aldehyde dehydrogenase	-	67160	1.5
87	Hypothetical protein	-	04070	0.5
88	Hypothetical protein	-	04070	1.1
91	Dihydrolipoamide dehydrogenase (EC 1.8.1.4)	*lpdA*	13270	1.0
92	dihydrolipoamide dehydrogenase (EC 1.8.1.4)	*lpdA*	13270	0.4
94	Putative Zn metallo-β lactamase/putative rhodanese domain-containing protein	-	02720	1.8

S4 Table Continued

Spot	Protein identity	Gene	ORF (RERY xxxxx)	Ratio D/S
97	Putative flavin amine oxidase	-	02670	1.4
98	Aspartate kinase (EC 2.7.2.4)	*ask*	35530	0.4
102	Putative flavin amine oxidase	-	02670	0.7
108	Isocitrate lyase (EC 4.1.3.1)	*aceA*	01250	0.8
110	Putative acyl-CoA dehydrogenase	-	66330	1.5
114	Enolase	*eno*	24350	1.0
116	Putative acyl-CoA dehydrogenase	-	66330	1.8
118	Putative methyltransferase	-	63250	0.9
119	Hypothetical protein	-	38010	0.8
120	Methanol dehydrogenase (acceptor) apoprotein (EC 1.1.99.37)	*thcE*	08730	0.1
121	Methionine adenosyltransferase (EC 2.5.1.6)	*metK*	40140	1.1
123	Fructose-1,6-bisphosphatase class 2	*glpX*	21280	1.6
124	Putative acyl-CoA dehydrogenase	-	06810	0.8
127	Putative acyl-CoA dehydrogenase	-	08290	1.1
129	Putative Sorbitol dehydrogenase	-	30100	0.2
130	Beta-ketothiolase BktB	*bktB*	37770	0.9
131	Succinate--CoA ligase subunit beta	*sucC*	25890	0.7
133	Putative 3-ketoacyl-CoA thiolase	-	06740	0.8
134	Mycofactocin system (heme/flavin dehydrogenase)	*mftD*	08480	0.3
135	Beta-ketothiolase BktB	*bktB*	37770	0.5
138	Beta-ketothiolase BktB	*bktB*	37770	1.1
139	Quinolinate synthetase type A (EC 2.5.1.72)	*nadA*	50200	1.2
143	Putative luciferase-like monooxygenase	-	02920	0.2

S4 Table Continued

Spot	Protein identity	Gene	ORF (RERY xxxxx)	Ratio D/S
144	NADPH-dependent curcumin reductase	*curA*	32210	0.8
145	Putative beta-lactamase	-	10130	0.4
146	Putative acyl-CoA dehydrogenase	-	06760	0.7
147	Putative adenosine deaminase	-	60780	0.6
148	Putative aspartate-semialdehyde dehydrogenase	*asd*	35520	0.7
150	2,3,4,5-tetrahydropyridine-2,6-dicarboxylate N-succinyl transferase	*dapD*	56400	0.8
152	Aspartate-semialdehyde dehydrogenase	*asd*	35520	0.1
154	Aspartate-semialdehyde dehydrogenase	*asd*	35520	0.3
157	Putative ATP-binding transporter	-	39460	1.9
158	Electron transfer flavoprotein alpha subunit apoprotein	*etfA*	63340	1.5
160	Putative thiosulfate sulfurtransferase (EC 2.8.1.1)	-	31860	0.9
163	Putative thiosulfate sulfurtransferase (EC 2.8.1.1)	-	31860	1.5
166	Citrate lyase subunit beta-like protein	-	64520	1.4
167	Translation elongation factor 1A (EF-1A/EF-Tu)	*tufA*	08860	1.6
169	Citrate lyase subunit beta-like protein	-	64520	1.1
171	Pyridoxal 5′-phosphate synthase	*pdxS*	55960	0.4
173	Putative fumarylacetoacetate hydrolase domain-containing protein	-	63630	0.7
174	Putative luciferase-like monooxygenase	-	05640	0.8
175	NAD(P)-binding domain-containing protein	-	56980	0.4
176	Putative 3-hydroxybutyryl-CoA dehydrogenase	-	11770	0.6
177	Electron transfer flavoprotein beta subunit	*etfB*	63330	1.0
178	Electron transfer flavoprotein beta subunit	*etfB*	63330	1.8
179	Putative encapsulating protein	-	03200	0.7
180	Putative phosphate transport system regulatory protein	-	31960	0.7

S4 Table Continued

Spot	Protein identity	Gene	ORF (RERY xxxxx)	Ratio D/S
181	Putative Zn metallo-β lactamase/putative rhodanese domain-containing protein	-	02720	1.6
182	Putative thiazole synthase	-	55300	0.7
183	Electron transfer flavoprotein beta subunit	*etfB*	63330	0.3
185	Putative tellurium resistance protein	-	67580	0.5
186	NAD(P)-binding domain-containing protein	*yhfk*	25340	0.4
189	Isochorismatase family protein	*yecD*	02630	0.2
190	Isochorismatase family protein	*yecD*	02630	0.2
192	Translation elongation factor 2 (EF-2/EF-G)	*fusA*	08870	0.5
201	Putative taurine catabolism dioxygenase	-	58710	1.7
202	Putative phosphoglycerate mutase	-	01700	1.1
204	ATP-dependent Clp protease proteolytic subunit 2	*clpP*	53530	0.7
207	Putative OsmC-like protein	-	02650	0.8
208	Putative OsmC-like protein	-	02650	0.7
209	Putative pyridoxamine 5'-phosphate oxidase	-	57780	0.6
210	Putative cold-shock DNA-binding protein family	*cspA1*	34650	0.6
212	Putative peptidyl-propyl-cis-trans isomerase binding protein	-	27670	0.9
213	Putative rhodanese-related sulfur transferase	-	02740	0.9
